# In Silico Studies of Potent Tyrosine Kinase Inhibitors: Molecular Docking and Pharmacophore Modeling Approaches

**DOI:** 10.3390/molecules31101689

**Published:** 2026-05-16

**Authors:** Evangelos Mavridis, Eleni Pontiki, Dimitra Hadjipavlou-Litina

**Affiliations:** Laboratory of Pharmaceutical Chemistry, School of Pharmacy, Faculty of Health Sciences, Aristotle University of Thessaloniki, 54124 Thessaloniki, Greece; eamavridi@pharm.auth.gr (E.M.); epontiki@pharm.auth.gr (E.P.)

**Keywords:** tyrosine kinase inhibitors, molecular similarity, molecular docking, pharmacophore modeling, virtual screening, compound repurposing

## Abstract

Compound repurposing is an efficient method to save both time and costs by redirecting previously synthesized small molecules towards new biological targets. In this research, we employ computational methodologies to investigate and assess target engagement of small molecules as tyrosine kinase inhibitors (TKIs). Therefore, compounds **TKI.2a**, **TKI.2b**, **TKI.6**, **TKI.16**, **TKI.19**, and **TKI.21b** identified from our earlier research, undergo assessments of molecular similarity, docking studies, and pharmacophore modeling along with those discovered through database searches. Compounds **TKI.2a**, **TKI.2b**, **TKI.6**, and **TKI.19** appear to exhibit multi-target tyrosine kinase inhibitory activities against VEGFR-2 (Vascular Endothelial Growth Factor Receptor), RET (proto-oncogene tyrosine–protein kinase receptor), PDGFRα (Platelet-Derived Growth Factor Receptor alpha), EGFR (Epidermal Growth Factor Receptor), and HER2 (Human Epidermal Receptor) receptors. Pharmacophore models were applied for ligand-based virtual screening using defined parameters to discover candidate compounds that exhibit drug-likeness with FDA (Food and Drug Administration)-approved tyrosine kinase inhibitors. Molecular docking studies identified lead compounds for each biological target based on their overall affinity values and established interactions. Compound **ChEMBL2170947** was found to be the most promising candidate for the VEGFR-2 receptor, **ChEMBL5019511** for PDGFRα, **ChEMBL2216869** for EGFR, and **ChEMBL3355044** for HER2.

## 1. Introduction

The application of computational methods in drug discovery began in the early 1960s, highlighted by the quantitative structure–activity relationship studies conducted by Prof. Corwin Hansch and his research group [1]. As more data was generated and became increasingly accessible, advanced algorithms were also developed to analyze it. Machine learning includes a wide variety of algorithms that enable systems to learn from data without direct programming, whereas deep learning specifically applies artificial neural networks with multiple layers to interpret data [2].

The application of machine learning (ML) in drug discovery represents a groundbreaking method showing a significant change in how scientists and researchers develop new therapeutic compounds. Prominent examples of this advancement include the implementation of Quantitative Structure–Activity Relationships (QSARs) and the prediction of pharmacokinetic/toxicity (ADMET—Absorption, Distribution, Metabolism, Excretion, Toxicity) properties, which made possible the prediction of biological activities based on molecular structure, considering that molecules with similar structures tend to exhibit comparable bioactivity [3]. Furthermore, the application of deep learning (DL) algorithms, such as convolutional neural networks, has improved the ability to recognize and evaluate intricate patterns and images within large datasets, for instance, in molecular docking studies [2].

In silico approaches offer a deeper understanding of atomistic details that current experimental methods cannot achieve. Naturally, the primary aim of computational methods is not to substitute experiments but to assist multidisciplinary scientific teams in applying more efficient and comprehensive strategies.

In this study, we employed in silico computational methods to explore and evaluate the bioactivity of small molecules that are newly developed as tyrosine kinase inhibitors (TKIs). The frequent deregulation of tyrosine kinases (TKs) in various diseases has inspired investigations into their structure. This structural arrangement is primarily characterized by the presence of two distinct lobes: the smaller N-terminal lobe (N-lobe) and the larger C-terminal lobe (C-lobe). Together, these lobes create a deep cleft that serves as the active site, which accommodates an ATP (Adenosine triphosphate) molecule in conjunction with either one or two divalent cations, such as magnesium or manganese [4]. The N-lobe and C-lobe are linked by an area known as the hinge region, which allows the relative movement of these two lobes. Such movements are essential for catalysis, as they spatially arrange the catalytic residues into a well-conserved configuration that enables the transfer of phosphate. In most kinases, the N-terminal lobe (N-lobe) of the protein kinase domain is typically characterized as a small, predominantly anti-parallel-sheet structure comprising five strands (labeled *β*1–*β*5) and a crucial regulatory single helix, the αC-helix. The αC helix is crucial for catalysis and for transitioning the kinase between its inactive and active forms. Additionally, the N-lobe features a significant glycine-rich loop that connects the *β*1 and *β*2 strands, known as the phosphate-binding loop (P-loop or G-loop). In contrast, the C-lobe is more stable and primarily composed of alpha helices. It includes seven helices (αD–αI) and two to four very short *β* strands (*β*6–*β*9). Throughout the catalytic cycle, an important structural element called the activation loop (A-loop) alters its conformation. The N-terminal section of the activation loop contains a segment featuring a highly conserved sequence, Asp-Phe-Gly, referred to as the DFG motif, which is involved in coordinating metal cations. The central region of the activation loop varies more between different kinases, both in length and sequence, and is involved in substrate binding, ensuring that the substrate is appropriately positioned for phosphorylation. The activation loop also includes one or more phosphorylation sites playing a key role in the kinase activation process [5]. It seems that these characteristics are essential for grasping kinase catalytic activity. However, in the process of designing an inhibitor, the main objective is to replicate certain aspects of ATP’s binding and to investigate additional areas where the synthetic compound can forge new interactions, focusing on selectivity [2].

In our earlier docking studies [6], we discovered that compounds **TKI.2a**, **TKI.2b**, **TKI.6**, **TKI.16**, **TKI.19**, and **TKI.21b** maintained their original biological targeting (Table 1).

**Figure 1:**
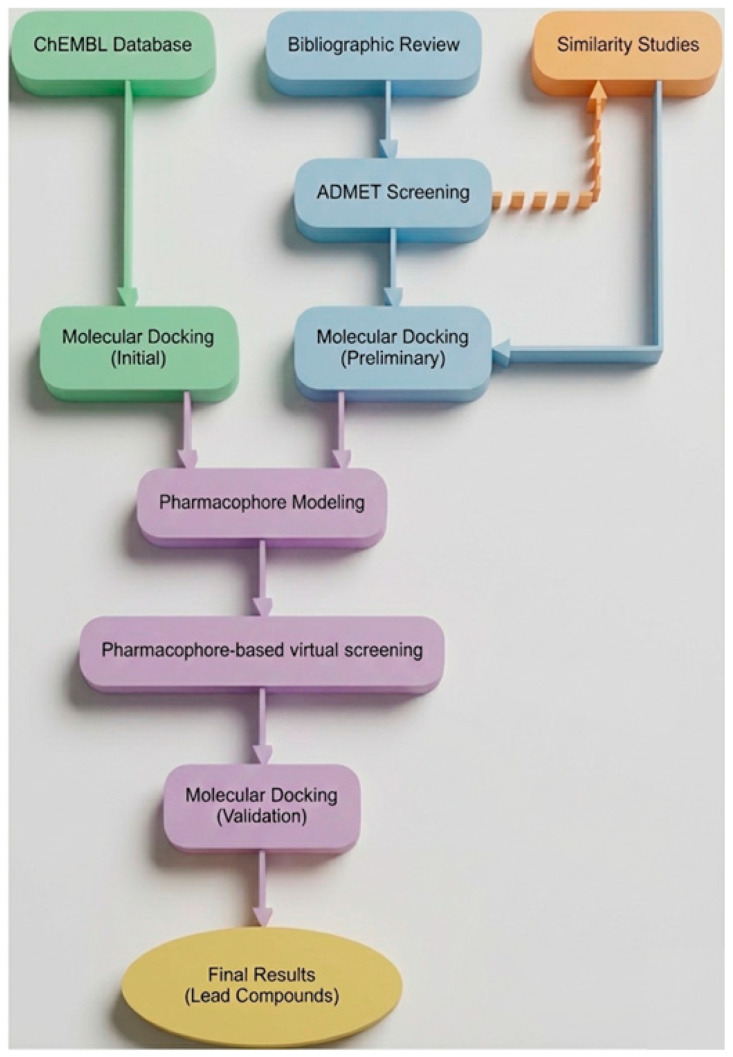
Flowchart displaying the procedure of our research.

Therefore, in the project herein, the aforementioned compounds underwent molecular similarity studies to identify new potential tyrosine kinase targets based on their structural similarity to known FDA (Food and Drug Administration)-approved drugs. New biological targets that emerged, along with the previously identified targets, were utilized in molecular docking studies (preliminary) for validation purposes (Figure 1).

All validated compounds were combined with standout molecules from the docking screening (initial) of the ChEMBL database [12,13] corresponding to each specific target. The newly organized groups underwent pharmacophore modeling, isolating pharmacophores for VEGFR-2, EGFR, HER2, and PDGFRα (Platelet-Derived Growth Factor Receptor alpha). Ultimately, these scaffolds were employed to screen the entirety of the ChEMBL34 database [14], leading to the identification of potent TKIs, which, following additional molecular docking studies (validation), could be classified as lead compounds against these specific biological targets.

## 2. Results and Discussion

### 2.1. Molecular Similarity

Descriptors are classified based on their dimensional complexity, a system that mirrors the richness of the features they extract. Higher-dimensional descriptors offer a more sophisticated look at a molecule’s geometry and interactions, whereas lower-dimensional ones focus on fundamental structural counts. Molecular weight and logP represent typical 1D descriptors, while topological indicators and fingerprints fall under the category of 2D descriptors. 3D descriptors encompass a wide variety of properties, including electrostatic potential and the three-dimensional arrangement of ligand moieties [2].

#### Molecular Similarity Studies

The Tanimoto coefficient (T) is the most widely used metric for evaluating the similarity of new compounds to existing FDA-approved drugs. Compounds that exceed a 90% similarity threshold [15], specifically those with *Tani_Atom pairs_* ≥ 0.237 and *Tani_MACCS_* ≥ 0.528 simultaneously, were considered eligible (Table S1) for targeting new biological entities, as molecules with comparable structures often show similar bioactivity [16]. The most noteworthy findings with the highest similarity indices were observed between **TKI.2a** and Tivozanib (VEGFR-2), which had *Tani_Atom pairs_* = 0.44 and *Tani_MACCS_* = 0.62; between **TKI.2b** and Tivozanib (VEGFR-2), with *Tani_Atom pairs_* = 0.48 and *Tani_MACCS_* = 0.75; and finally, between **TKI.21b** and Capmatinib (c-MET/HGFR—hepatocyte growth factor receptor), where *Tani_Atom pairs_* = 0.27 and *Tani_MACCS_* = 0.63. Compounds were investigated to meet similarity criteria with known FDA-approved drugs. All the potential investigated biological targets for the compounds that follow the FDA-approved drugs molecular similarity criteria are given in Table 2.

It is striking that the shared urea group among **TKI.2a**, **TKI.2b**, and Tivozanib correlated with their elevated Tanimoto indices. Further clarification of additional common substructures between the compounds and the drugs could be achieved through pharmacophore modeling studies.

Newly identified biological targets include **c-Kit/SCFR**, **PDGFRα**, **MEK1/2**, **RET, JAK1/2**, and **c-MET/HGFR**, many of which are undergoing molecular docking studies with our compounds to validate the results of molecular similarity assessments or to explore new ones.

### 2.2. Molecular Docking

Molecular docking focuses on modeling the interaction between a molecule and the three-dimensional structure of a specific target. The main aim of docking is to identify a favorable conformation of the target–ligand complex, enhancing the number of interactions while reducing the predicted binding energy (calculated from the scoring function). Scoring functions (SFs) are computational algorithms employed in molecular docking simulations to evaluate and rank various ligand conformations within the target protein [2].

#### 2.2.1. Statistical Analyses

Binding efficacy was evaluated using selection criteria established through statistical analyses of molecular docking results for FDA-approved drugs and their reported biological targets [17]. The criteria included (i) binding affinity measured in kcal/mol, (ii) CNN (convolutional neural network) pose score, which estimates the likelihood that the pose exhibits minimal root mean square deviation (RMSD) from the reference binding pose, and (iii) CNN affinity, representing the predicted affinity to the biological target as determined by the convolutional neural network.

Table 3 presents the average values of the parameters affinity, CNN pose score, and CNN affinity for each biological target, along with the overall averages, maximum, and minimum values. The CNN pose score ranges from 0 to 1, where a value of 1 indicates a high probability that the ligand’s conformation within the cavity is optimal. Inferential statistical analyses were conducted at a significance level of α = 0.05 (95% confidence limits) to determine the upper confidence limit for affinity and the lower confidence limits for CNN pose score and CNN affinity, as detailed below:

- One-sided upper confidence interval (CI) for the affinity parameter: (−∞, −9.00);
- One-sided lower confidence interval (CI) for the CNN pose score parameter: (0.843, ∞);
- One-sided lower confidence interval (CI) for the CNN affinity parameter: (7.702, ∞).

Statistical analysis revealed that the estimated 95% confidence limits (soft thresholds) for affinity (−9.00), CNN pose score (0.843), and CNN affinity (7.702) significantly surpassed their respective benchmark values (hard thresholds) of −6.82, 0.526, and 7.010 (Table 3). As these benchmarks fall outside the one-sided confidence intervals, the null hypothesis was rejected in each case. This provides 95% confidence that the “true/real means” of potent TKIs’ population lies within the calculated limits.

The results of the molecular binding studies were evaluated according to the following criteria:

(i) They must surpass at least two of the three soft thresholds determined through statistical analyses;
(ii) If a soft threshold is not achieved, the value must not fall below or rise above the corresponding minimum or maximum value (hard thresholds), respectively;
(iii) The interactions between the chemical groups of the compounds and those of the receptors should always be considered, alongside the interactions given in the literature [18].

Only the results that satisfied the criteria (i) and (ii) are discussed below and we investigated compounds’ interactions per biological target.

#### 2.2.2. Vascular Endothelial Growth Factor Receptor 2 (VEGFR-2)

Due to the fact that the DFG (Asp-Phe-Gly) residues are positioned to impede ATP binding and obstruct the substrate binding site, the selected X-ray crystal structure of VEGFR-2 (PDB ID: 4ASE–Tivozanib) [19] displays a “DFG-out” (inactive) conformation, signifying that the kinase is predominantly inactive. The DFG motif area, the hinge region, and the hydrophobic regions (HYD-I and HYD-II) constitute the four main sections of the VEGFR-2 active site.

**Figure 2:**
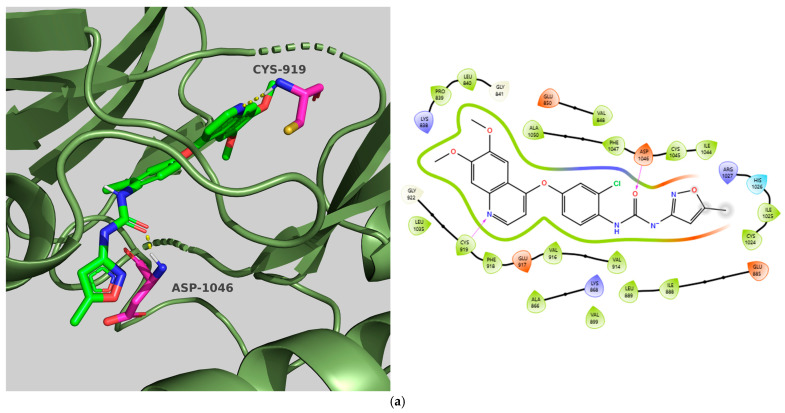

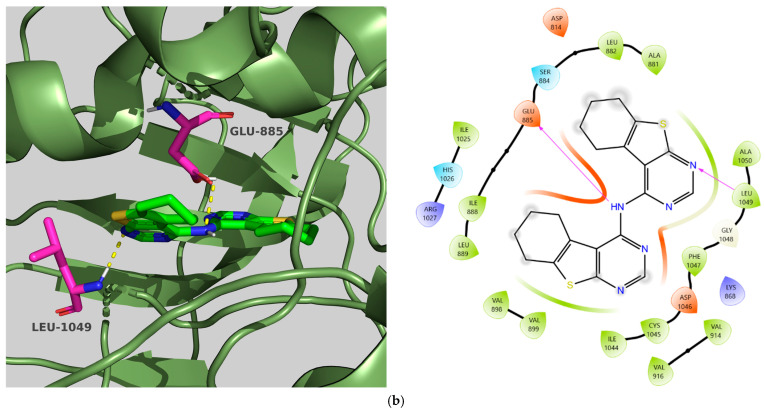
Preferred docking pose (3D) and ligand interaction diagram (2D) of (**a**) Tivozanib; (**b**) TKI.6, with VEGFR-2 (PDB ID: 4ASE). Green core: ligands; magenta core: key amino acids; yellow dashes/magenta arrows: hydrogen bond interactions.

Regarding the docking data, **TKI.6** demonstrated binding and CNN affinities of −11.13 and 7.932, respectively. These values are comparable to those of Tivozanib, the co-crystallized ligand, possessing a binding affinity of −10.87 kcal/mol and a CNN affinity of 8.124. **TKI.6** reported a CNN pose score of 0.667, which is considerably lower than Tivozanib’s 0.925. Once redocked in its co-crystal form, the ligand Tivozanib formed two hydrogen bonds: one with the DFG domain’s Asp1046 and another with the hinge area at Cys919. Hydrophobic interactions were observed in the HYD-I region with Leu840 and Phe918 as well as in the more specific HYD-II region featuring Ile888, Leu889, Val899, and the gatekeeper residue Val916. Conversely, **TKI.6** established an H-bond with Glu885 (αC helix) and yet showed no interaction with the hinge region (Cys919). **TKI.6’s** unsatisfactory performance in the CNN pose score (<0.843 one-sided lower CI) might be explained by this deviation. Additionally, hydrophobic interactions were observed with Glu885 and Asp1046, along with the HYD-II region (Val898). Finally, **TKI.6** exhibited an extra H-bond interaction with Leu1049 (Figure 2).

#### 2.2.3. Proto-Oncogene Tyrosine–Protein Kinase Receptor (RET)

In our study of RET kinase inhibitors, we employed the RET protein tyrosine kinase in association with Pralsetinib (PDB ID: 7JU5) [20]. This structure features a hinge region spanning from Glu805 to Ala807 and a hydrophobic pocket situated between the gatekeeper residue Val804 and the catalytic lysine Lys758. The gatekeeper pocket is recognized for its role in influencing the affinity and selectivity of numerous classes of kinase inhibitors [21].

**Figure 3:**
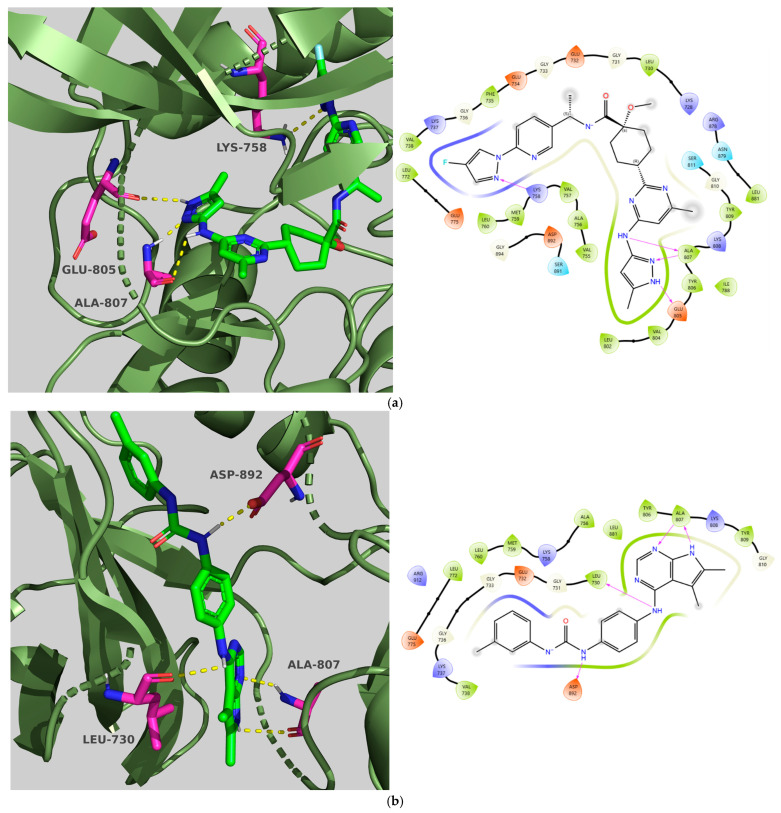
Preferred docking pose (3D) and ligand interaction diagram (2D) of (**a**) Pralsetinib; (**b**) TKI.2a, with RET (PDB ID: 7JU5). Green core: ligands; magenta core: key amino acids; yellow dashes/magenta arrows: hydrogen bond interactions.

Pralsetinib showed three hydrogen bonds with the hinge region (Glu805, Ala807, Ala807), enhancing its binding to the protein (affinity: −9.89, CNN pose score: 0.970 and CNN affinity: 8.129). In contrast, **TKI.2a** lost one hydrogen bond connection (Ala807, Ala807), which may explain the lower CNN affinity score (affinity: −9.87, CNN pose score: 0.866 and CNN affinity: 7.349) when compared to the co-crystallized ligand. Additionally, another distinction is that Pralsetinib formed a hydrogen bond with the catalytic lysine Lys758, while **TKI.2a** formed bonds with the DFG domain’s Asp892 and Leu730 of the b1 strand on the N-lobe side. Lastly, regarding hydrophobic interactions, both ligands stabilized within the selectivity pocket by interacting with residues Leu730 and Val738 located on the b2 strand (glycine-rich loop) on the N-lobe side, along with the catalytic lysine Lys758, gatekeeper Val804, and Leu881 from the b7 strand on the C-lobe side [20] (Figure 3).

#### 2.2.4. Platelet-Derived Growth Factor Receptor Alpha (PDGFRα)

Compounds **TKI.2a**, **TKI.2b,** and **TKI.19** demonstrated significant outcomes in molecular docking experiments conducted using the X-ray crystal structure of PDGFRα (PDB ID: 6JOL) [22]. The key residues in this structure include a hinge region residue (Cys677), a gatekeeper residue (Thr674), a catalytic loop residue (Val815), residues of the DFG motif (Asp836, Phe837), and a residue from the αC-helix (Glu644). Ultimately, interactions with hydrophobic residues such as Val607, Met648, Val658, Leu825, Cys835, and Phe837, along with the occupation of a hydrophobic pocket located between the catalytic loop and αC-helix (Met648, Ile657, Val815, Leu809, Ile834), may play a vital role in enhancing activity against PDGFRα [22].

**Figure 4:**
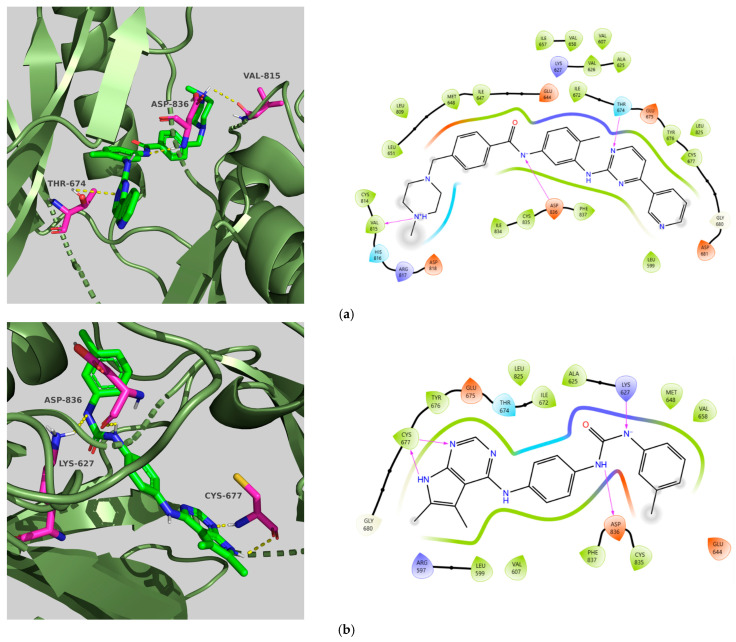

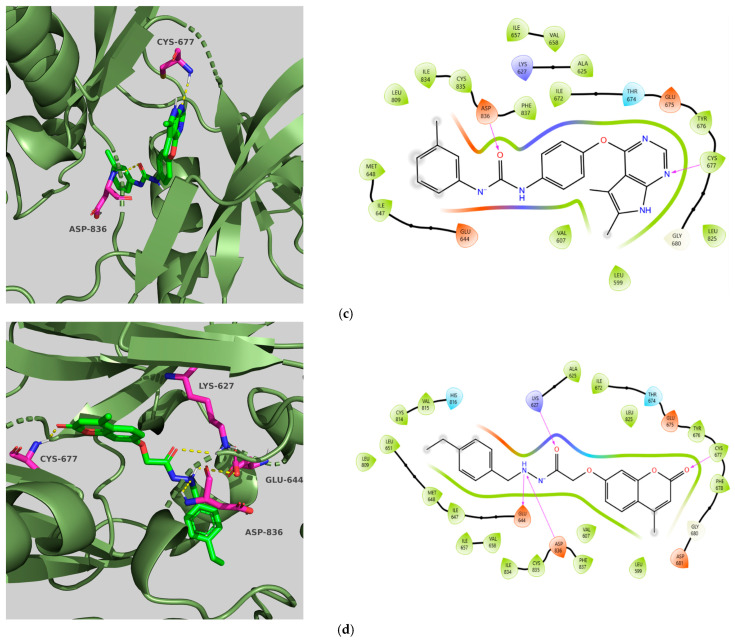
Preferred docking pose (3D) and ligand interaction diagram (2D) of (**a**) Imatinib; (**b**) TKI.2a; (**c**) TKI.2b; (**d**) TKI.19, with PDGFRα (PDB ID: 6JOL). Green core: ligands; magenta core: key amino acids; yellow dashes/magenta arrows: hydrogen bond interactions.

Compounds **TKI.2a** and **TKI.2b** exhibited the most favorable outcomes (CNN pose score: 0.963, 0.977 and CNN affinity: 7.936, 8.108, respectively) by establishing hydrogen bonds with the hinge region and the DFG motif. The most significant distinction in comparison with Imatinib and **TKI.19**, which enhanced both CNN affinity and CNN pose score values, appeared to be the shared hydrophobic interactions with residues Glu644, Val658, Leu825, and Phe837. Notably, the hydrophobic interactions with Phe837 of the DFG motif were achievable due to the Asp-Phe-Gly/DFG-out conformation characteristic of the inactive form of PDGFRα. Even though Imatinib lost a crucial hydrogen bond with the hinge region, it still maintained a high CNN affinity and CNN pose score (7.952 and 0.862, respectively), possibly due to the establishment of two hydrogen bonds with the gatekeeper residue Thr674 and catalytic Val815, along with interactions with several hydrophobic residues (Leu599, Val607, Ala625, Lys627, Ile672, Thr674, Leu825, Asp836). Compound **TKI.19** exhibited four hydrogen bonds (Lys627, Glu644, Cys677, and Asp836) yet paradoxically had a CNN affinity of only 7.040. A possible explanation for this could be that **TKI.19** had the lowest hydrophobic interactions (Leu599, Val607, Ala625, Leu809, Asp836) compared to other compounds. Ultimately, the elevated CNN pose score (0.872) was attributed to its distinct double engagement with Leu809 in the hydrophobic pocket (Figure 4).

#### 2.2.5. Epidermal Growth Factor Receptor (EGFR)

In our molecular docking study, we dealt with a member of the HER (Human Epidermal Growth Factor Receptor) kinase family, specifically EGFR. To facilitate this, we employed the surrogate crystal structure of the wild-type EGFR bound to mobocertinib (PDB ID: 7T4I) [23]. This study emphasizes key residues, such as Met793 located in the hinge region, and particular areas like the selectivity pocket, where Thr790 acts as the gatekeeper for ATP binding, Lys745 functions as the catalytic residue, and Thr854 interacts with the DFG motif (855–857), in addition to two distinct hydrophobic regions. Hydrophobic region I includes amino acids like Phe723, Leu747, Ile759, Met766, Leu777, and Leu788, while hydrophobic region II, found near Thr790 and made up of Leu718, Gly719, Val726, and Leu844, is vital for binding compounds to EGFR. Finally, Cys797, located at the border of the active site cleft is recognized as the most solvent-exposed cysteine in the EGFR kinase domain, being a key to forming covalent bonds with irreversible TKIs.

The binding affinities of the selected compounds, **TKI.2a** (−8.60) and **TKI.19** (−8.17), outperformed the co-crystallized ligand mobocertinib (−7.66), even though they do not reach the conventional −9.00 soft threshold. The literature suggests that molecular docking frequently underestimates the affinity of FDA-approved drugs for wild-type EGFR [24,25]. This is primarily attributed to the inability of docking models to account for EGFR’s significant conformational shifts [26]. Consequently, a category-specific threshold of −7.95—representing the average affinity of FDA-approved inhibitors—was established (Table 3). Notably, CNN scoring bypassed these docking limitations, providing a more accurate representation of binding parameters, demonstrating superiority over traditional affinity metrics.

**Figure 5:**
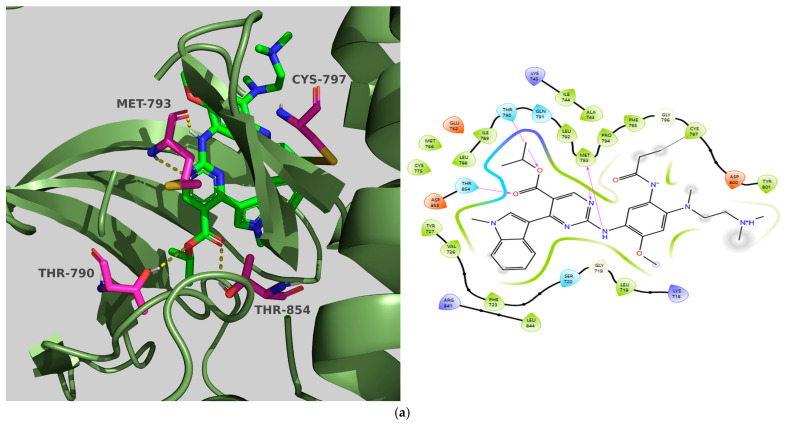

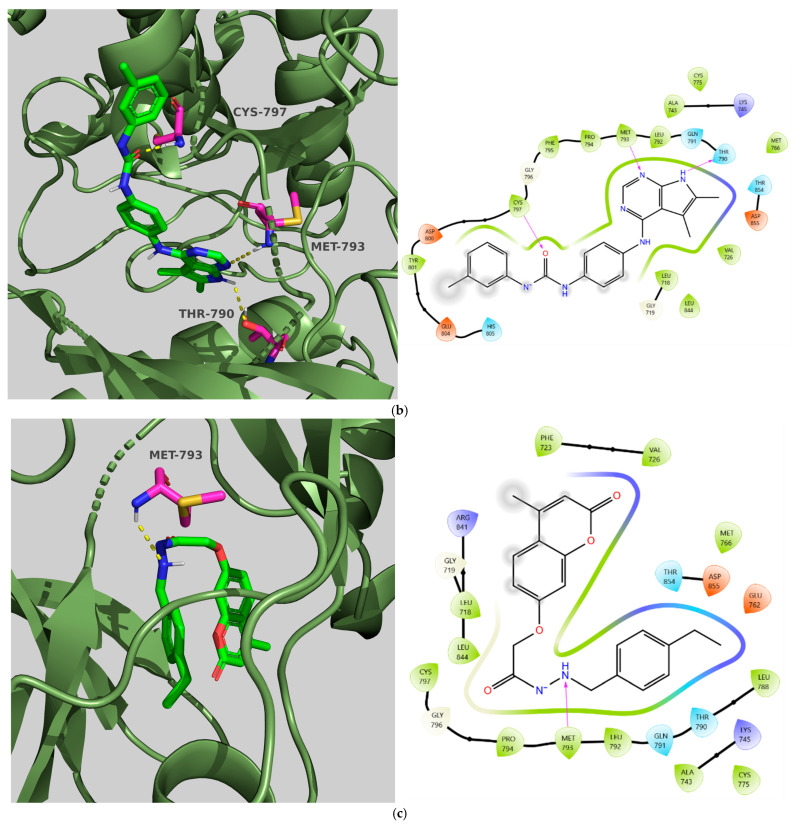
Preferred docking pose (3D) and ligand interaction diagram (2D) of EGFR with (**a**) mobocertinib; (**b**) TKI.2a; (**c**) TKI.19, showing key interactions at the active site of EGFR (PDB ID: 7T4I). Green core: ligands; magenta core: key amino acids; yellow dashes/magenta arrows: hydrogen, black lines: covalent bonds.

Both the co-crystallized ligand and the chosen compounds established hydrogen bonds with the hinge region. However, mobocertinib exhibited a greater affinity than **TKI.2a** and **TKI.19** for the active site, attributed to an additional hydrogen bond with Met793, another bond with the gatekeeper Thr790, and with Thr854, and primarily due to a covalent bond with Cys797, which explains the differences in their CNN affinities of 8.106, 7.047, and 7.087, respectively. In contrast, **TKI.2a** formed two hydrogen bonds with the gatekeeper Thr790 and Cys797, along with hydrophobic interactions with region II (Leu718, Val726, and Leu844), resulting in a CNN pose score of 0.843, while mobocertinib achieved a score of 0.970. Finally, **TKI.19**, despite forming only one hydrogen bond with Met793, was appropriately positioned in the binding site due to extensive hydrophobic interactions with hydrophobic region I (Phe723, Leu788), key region II (Val726, Leu844), and the gatekeeper residues Thr790 and Thr854, leading to a remarkable CNN pose score of 0.851 (Figure 5).

#### 2.2.6. Receptor Tyrosine–Protein Kinase erbB-2 (HER2)

**Figure 6:**
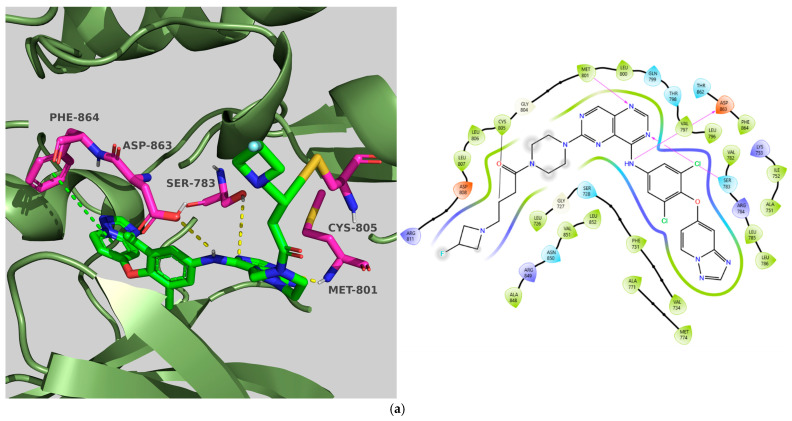

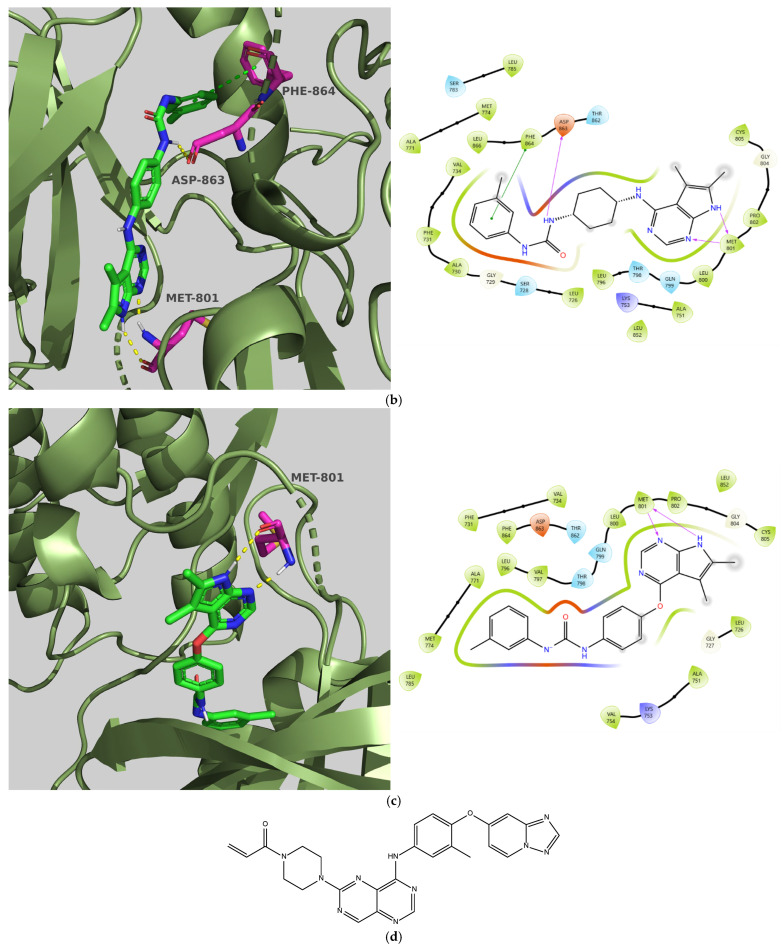
Preferred docking pose (3D) and ligand interaction diagram (2D) of (**a**) covalent inhibitor; (**b**) TKI.2a; (**c**) TKI.2b, showing key interactions at the active site of HER2 (PDB ID: 7PCD). (**d**) Chemical structure of BI-1622, covalent inhibitor’s close analog. Green core: ligands; magenta core: key amino acids; yellow dashes/magenta arrows: hydrogen bond interactions; green dashes/green lines: π-stacking; black lines: covalent bonds.

The selected X-ray crystal structure of HER2 (PDB ID: 7PCD–covalent inhibitor) [27] displays the typical bilobed folding pattern found in kinases. The glycine-rich nucleotide phosphate-binding loop (P-loop;Leu726–Val734) and the αC helix (Pro761–Ala775) of the N-lobe are connected by a flexible hinge region (Met801) to the DFG motif (Asp863–Gly865), the catalytic loop (Arg844–Asn850), and the activation loop (A-loop; Asp863–Val884) of the C-lobe of the kinase, and divided by a deep cleft that contains the ATP binding site [28]. The covalent inhibitor (IUPAC name: 1-[4-[4-[[3,5-dichloro-4-([1,2,4]triazolo[1,5-a]pyridin-7-yloxy)phenyl]amino]pyrimido[5,4-d]pyrimidin-6-yl]piperazin-1-yl]-4-(3-fluoroazetidin-1-yl)but-3en-1-one) is a close analog of BI-1622 (Figure 6d), a lead compound developed by Boehringer Ingelheim as a proof-of-concept molecule for selective HER2 inhibition [27].

The docking studies of the co-crystallized ligand (covalent inhibitor) indicated that a prominent hydrogen bond was established in the hinge region with Met801, in addition to a hydrogen bond and a π-stacking interaction involving Asp863 and Phe864, which are both components of the DFG motif. Although significant hydrophobic interactions were lacking, leading to a lower CNN pose score (0.814), a covalent bond with Cys805 resulted in an increased CNN affinity value (7.734). In contrast, **TKI.2b** exhibited a notably higher CNN pose score (0.885) and a satisfactory CNN affinity score (7.410) due to a wealth of hydrophobic interactions (Leu726, Leu726, Phe731, Val734, Lys753, Ala771, Leu785, Leu796, Leu796, Leu852, Phe864) and two hydrogen bonds established in the hinge region (Met801). Ultimately, **TKI.2a**, which formed three hydrogen bonds (two with Met801 and one with Asp863) and a π-stacking interaction with Phe864, along with multiple hydrophobic interactions (Leu726, Leu726, Val734, Lys753, Leu796, Leu852, Thr862), achieved the highest CNN pose and CNN affinity values of 0.889 and 7.740, respectively. Finally, while Ser783 is regarded as a key amino acid that influences the selectivity between HER2 and EGFR activity, it is the only covalent inhibitor (co-crystallized) that demonstrated a hydrogen bond interaction (Figure 6).

#### 2.2.7. Hepatocyte Growth Factor Receptor (c-MET)

We employed the X-ray crystal structure of c-Met co-crystallized with Tepotinib (PDB ID: 4R1V), which reveals a DGF-in conformation, to investigate into binding mode [29]. Key characteristics of the complex include a fully resolved A-loop that extends into the ATP pocket, facilitating π-stacking interactions between potential inhibitors and Tyr1230. Nevertheless, while the interaction with the A-loop residue Tyr1230 is crucial for overall potency and selectivity, anchoring the molecule in the binding pocket through the hinge residue (Met1160) is equally significant [30]. Interactions with the DFG motif (Asp1222) and various hydrophobic residues (Ile1084, Val1092, Ala1108, Met1211, and Tyr1230) enhance the affinity values [31].

**Figure 7:**
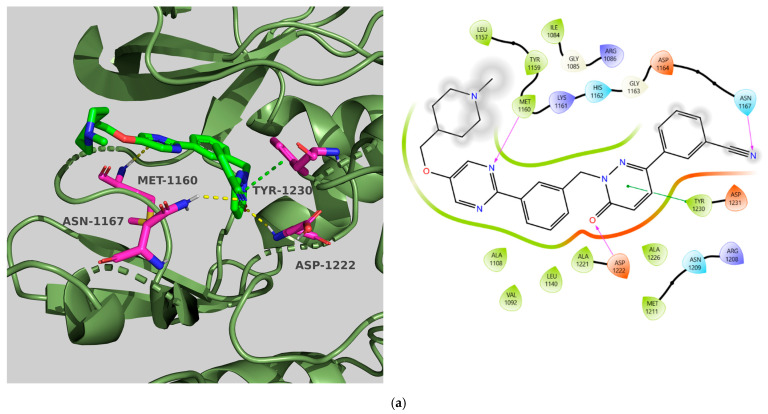

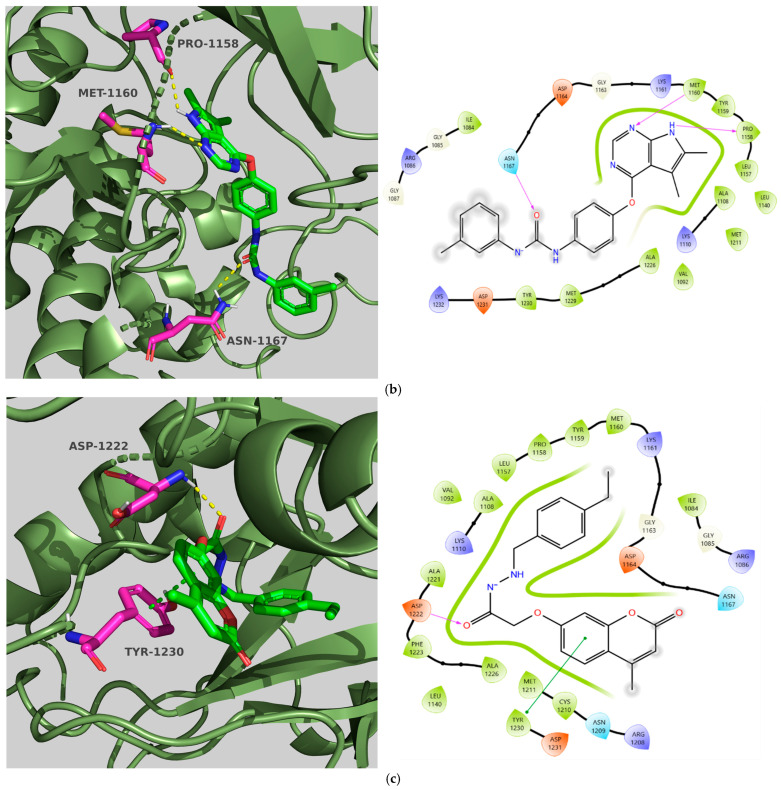
Preferred docking pose (3D) and ligand interaction diagram (2D) of (**a**) Tepotinib; (**b**) TKI.2b; (**c**) TKI.19, showing key interactions at the active site of c-MET (PDB ID: 4R1V). Green core: ligands; magenta core: key amino acids; yellow dashes/magenta arrows: hydrogen bond interactions; green dashes/green lines: π-stacking.

Tepotinib exhibited all essential interactions with the protein, including a hydrogen bond with the hinge region and π-stacking with Tyr1230, along with two hydrogen bonds to Asp1222 and Asn1167, which contributed to its high affinity scores (affinity: −10.00, CNN pose score: 0.863, and CNN affinity: 8.222). **TKI.2b** and **TKI.19** were unable to meet the CNN affinity lower confidence limit (7.120 and 7.199 < 7.702), despite demonstrating key interactions with either Met1160 or Tyr1230 that allowed for proper orientation in the binding pocket, maintaining high CNN pose scores at 0.843 and 0.856, respectively. The compound **TKI.2b** formed two additional hydrogen bonds with Pro1158 and Asn1167, while **TKI.19** established a hydrogen bond with Asp1222. All three compounds showed beneficial hydrophobic interactions with the residues (Val1092, Leu1157, Tyr1230), with **TKI.19** demonstrating two additional interactions with Ile1084 and Ala1108, compensating for the fewer hydrogen bond interactions in comparison with the other two compounds (Figure 7).

In conclusion, a greater number of hydrophobic interactions enhance CNN pose scores, alongside binding with hinge and gatekeeper residues. Additionally, the affinity of CNN is primarily influenced by the total number of interactions as well as their quality. Therefore, interactions with particular hydrophobic residues that are vital for binding, an increased number of hydrogen bonds with hinge and gatekeeper residues, covalent interactions, and π-stacking appear to strengthen CNN affinity. All interactions and binding affinities are thoroughly outlined in Table S2. The segments of molecules that are engaged with proteins are identified in pharmacophore modeling studies (Section 2.3).

The docking studies results indicate that the compounds presented in Table 4 in addition to their initially identified biological target(s), might be inhibitors for additional kinases.

#### 2.2.8. Validation Results

It is important that there is not any universally applicable validation technique for docking studies, as the selection of a validation approach is contingent on the specific context, research goals, and the available data. Often, a variety of validation methods are employed to achieve a thorough evaluation of the performance of docking techniques. In this study, we utilized standard methods for evaluating the accuracy of docking protocols, which included self-docking, cross-docking, and ligand enrichment.

The docking procedure was initially validated by re-docking the co-crystallized ligand in the vicinity of the enzyme’s binding site and, subsequently, calculating the root mean square deviation (RMSD) between the final conformation and the original coordinates. RMSD values below 2.0 Å indicate consistent results; values ranging from 2.0 Å to 3.0 Å suggest a deviation from the reference position while maintaining the intended orientation. RMSD values beyond 3.0 Å are deemed completely inaccurate [18]. In our molecular docking studies, we recorded reliable RMSD values of 1.144 Å for VEGFR-2, 1.171 Å for RET, 1.417 Å for PDGFRα, 1.430 Å for EGFR, 1.121 Å for HER2, and 0.900 Å for c-MET, all of which stayed below 1.5 Å.

To further assess the docking protocols, cross-docking procedures were implemented. In the cross-docking analyses, each known FDA-approved ligand of the specified biological targets was docked into the above-mentioned receptors. According to Table 5, a significant majority (20 out of 25) of the known drugs achieved measurements of below 3 Å, confirming the reliability of our docking protocols. The only exceptions were Axitinib, Pazopanib, and Sunitinib in their molecular studies on VEGFR-2, Lenvatinib on RET, and Avapritinib on PDGFRα, which exhibited measurements of greater than 3 Å.

Finally, to assess the docking program, we employed the enrichment factor (*EF*), which acts as an indicator of the docking program’s trustworthiness. The objective was to evaluate the ability of the receptor to differentiate between inactive substances and known active compounds by determining enrichment values. An enrichment factor (*EF*) exceeding 1 demonstrates that the approach is more efficient than random selection, with higher values indicating improved performance. For instance, an *EF* of 5 in the top 5% of the dataset implies that there are five times more active compounds present in that top 5% of the evaluated set than one would anticipate by random chance.

**Figure 8:**
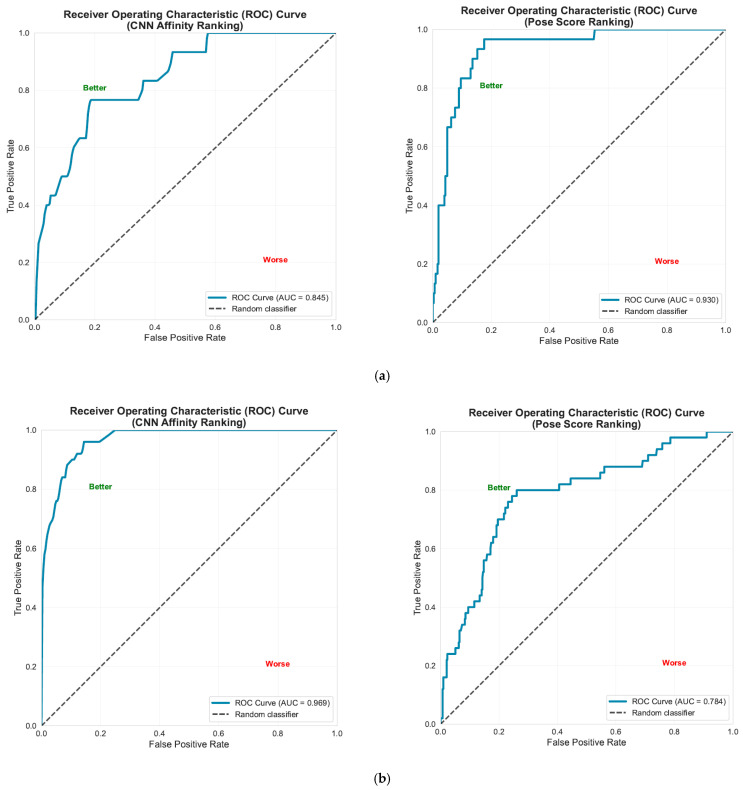
Receiver operating characteristic (ROC) curve and area under the ROC curve (AUC-ROC) ranked by CNN affinity and CNN pose score for (**a**) HER2; (**b**) VEGFR-2 proteins.

In this study, we used two proteins as receptors: HER2 (PDB ID: 3PP0) and VEGFR-2 (PDB ID: 2P2I). As far as HER2 is concerned, we analyzed a dataset consisting of 332 compounds, which included 30 active compounds and 302 inactive ones. As a result, when we ranked the compounds based on their CNN affinity values, the enrichment factor at 5% (*EF(5%)*) was calculated to be 6.917, successfully identifying 10 active compounds within the top 16 structures (representing 5%) (Supplementary Materials, Tables S3 and S4), which matched the CNN pose score enrichment factor at 5%. Furthermore, the receiver operating characteristic (ROC) curve along with the area under the curve (AUC) offered significant insights into the model’s ability to distinguish between active and inactive compounds across different threshold settings, with the AUC reflecting the model’s overall performance, as shown in Figure 8a. It is clear that the ranking based on the CNN pose score demonstrated a superior ROC-AUC compared to the CNN affinity ranking, showing values of 0.930 and 0.845, respectively. In the second scenario (VEGFR-2), we analyzed a total of 484 compounds, which included 50 active compounds. The CNN affinity enrichment factor was determined to be 9.277, significantly higher than that of the CNN pose score enrichment factor (*EF(5%)* = 4.840) (Supplementary Materials, Tables S5 and S6). Consequently, the CNN affinity exhibited a much better ROC-AUC (0.969) in comparison to the CNN pose score ranking (0.784), as depicted in Figure 8b.

Finally, after applying combined validation methods, we demonstrated that our molecular docking studies were able to yield reliable results.

### 2.3. Pharmacophore Modeling

As previously mentioned, 3D descriptors encompass a wide variety of properties, including electrostatic potential and the three-dimensional arrangement of ligand moieties, making them an essential component of pharmacophore modeling. Per IUPAC guidelines, a pharmacophore is defined as “an ensemble of steric and electronic features that is necessary to ensure the optimal supramolecular interactions with a specific biological target and to trigger (or block) its biological response” [2]. Consequently, pharmacophore methods are among the most valuable and significant tools developed, as they identify the molecular functional characteristics required for a molecule to bind to a specific receptor, subsequently guiding the virtual screening of extensive libraries of compounds to select the most suitable candidates [32].

Herein, we conducted pharmacophore modeling investigations for four biological targets (VEGFR-2, PDGFRα, EGFR, and HER2) to identify the key characteristics shared among active compounds that target each biological entity. The dataset of active compounds was compiled from substances sourced from the ChEMBL database following initial molecular docking, along with those identified from preliminary molecular docking results (Figure 1).

#### 2.3.1. Vascular Endothelial Growth Factor Receptor 2 (VEGFR-2)

**Figure 9:**
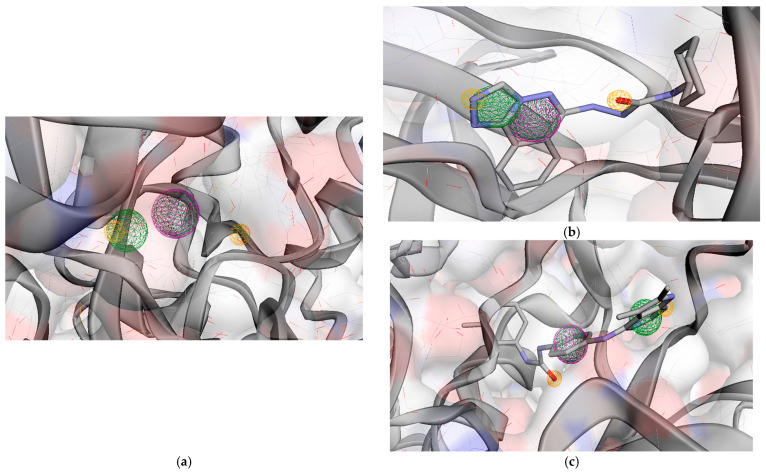
(**a**) A depiction of pharmacophore modeling for the VEGFR-2 receptor (PDB ID: 4ASE) demonstrates overall interaction features, comprising two hydrogen bond acceptors (HAC), two hydrophobic areas (HPB), and one aromatic component (ARO); (**b**) pharmacophore model aligned with the 16–VEGFR-2 interaction; (**c**) pharmacophore model aligned with the 2a–VEGFR-2 interaction. Hydrogen Acceptor: color = orange/radius (Å) = 0.5, hydrophobic: color = green/radius (Å) = 1.0, aromatic: color = purple/radius (Å) = 1.1.

The dataset consisted of 28 compounds that were effectively bound to the receptor, as demonstrated by molecular docking studies. The model achieved a performance score of 0.6628, demonstrating its effectiveness in integrating all individual molecular features. Analyses identified a five-feature pharmacophore map consisting of two hydrogen bond acceptors (HAC), two hydrophobic regions (HPB), and one aromatic ring (ARO) (Figure 9a). Generally, considering the positioning of these pharmacophores in relation to their role in ligand–receptor interactions (Figure 9b,c), the aromatic–hydrophobic groups appear to interact with the HYD-II region, while the basic hydrophobic one interacts with HYD-I. Furthermore, the HAC pharmacophore (located near the basic hydrophobic one) forms an essential bond with the hinge amino acid, and the second HAC interacts with the DFG region (Section 2.2.2).

#### 2.3.2. Platelet-Derived Growth Factor Receptor Alpha (PDGFRα)

**Figure 10:**
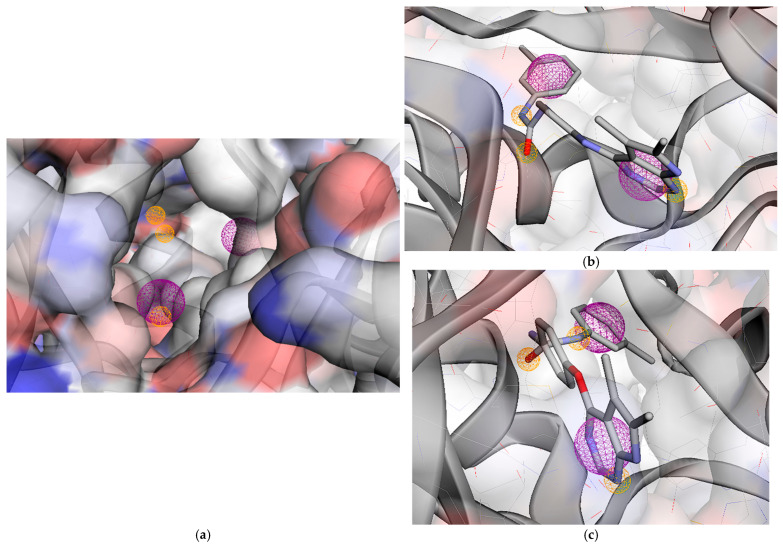
(**a**) A depiction of pharmacophore modeling for the PDFGRα receptor (PDB ID: 6JOL) demonstrates overall interaction features, comprising three hydrogen acceptors (HAC), and two aromatic groups (ARO); (**b**) pharmacophore model aligned with the 2a–PDFGRα interaction; (**c**) pharmacophore model aligned with the 2b–PDFGRα interaction. Hydrogen Acceptor: color = orange/radius (Å) = 0.5, aromatic: color = purple/radius (Å) = 1.1.

Molecular docking studies indicated that just five of the substances within the dataset were successfully bound to the receptor. Five pharmacophores were detected by the model’s performance, which was assessed at 0.8286, including two aromatic groups (ARO) and three hydrogen acceptors (HAC). The aromatic group and the attached HAC pharmacophore generally form essential bonds with the hinge and the gatekeeper residues. In contrast, the most active part of the model, composed of two HACs and an aromatic group, engages with the DFG region and the hydrophobic pocket between the catalytic loop and αC-helix (Figure 10) (Section 2.2.4).

#### 2.3.3. Epidermal Growth Factor Receptor (EGFR)

**Figure 11:**
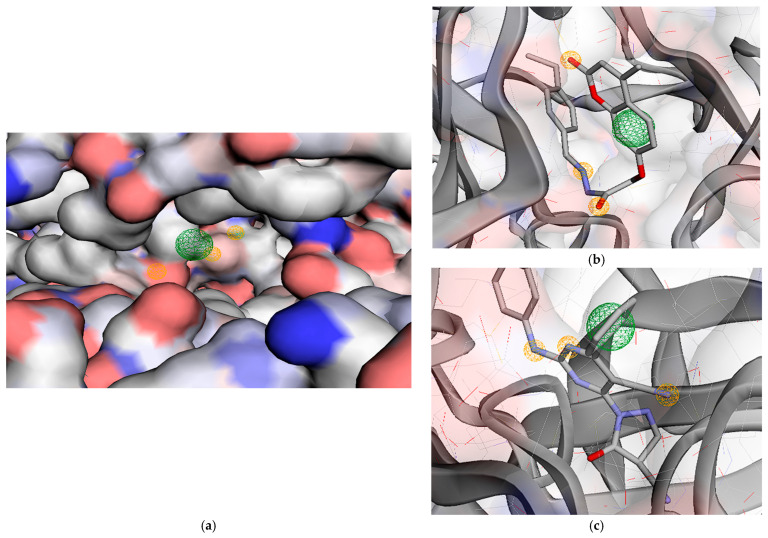
(**a**) A depiction of pharmacophore modeling for the EGFR (PDB ID: 7T4I) demonstrates overall interaction features, comprising three hydrogen acceptors (HAC), and one hydrophobic region (HPB); (**b**) pharmacophore model aligned with the 19–EGFR interaction; (**c**) pharmacophore model aligned with the 21b–EGFR interaction. Hydrogen acceptor: color = orange/radius (Å) = 0.5; hydrophobic: color = green/radius (Å) = 1.0.

The dataset comprised 32 compounds that were successfully bound to the receptor, as evidenced by molecular docking studies. Three hydrogen acceptors (HAC) and one hydrophobic group (HPB) were among the four pharmacophores identified by the model’s performance, which was assessed at 0.7707. The two HACs located near one another are responsible for interactions with the hinge region, while the distinct HAC is likely engaged with the DFG motif region. Finally, the interaction with the hydrophobic region II, which is essential for the binding of substances to EGFR, is facilitated by the hydrophobic group (Figure 11) (Section 2.2.5).

#### 2.3.4. Receptor Tyrosine–Protein Kinase erbB-2 (HER2)

**Figure 12:**
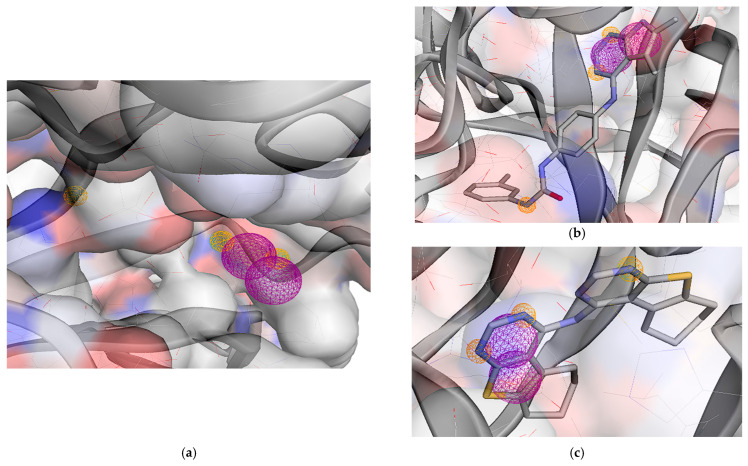
(**a**) A depiction of pharmacophore modeling for the HER2 receptor (PDB ID: 7PCD) demonstrates overall interaction features, comprising three hydrogen acceptors (HAC), and two aromatic groups (ARO); (**b**) Pharmacophore model aligned with the 2a–HER2 interaction; (**c**) Pharmacophore model aligned with the 6–HER2 interaction. Hydrogen Acceptor: color = orange/radius (Å) = 0.5, Aromatic: color = purple/radius (Å) = 1.1.

After conducting molecular docking studies, 12 compounds were analyzed to create a pharmacophore model with a reliability value of 0.8145, which includes three hydrogen bond acceptors (HAC) and two aromatic groups (ARO). The two adjacent HACs facilitate interaction with the hinge region, while the third HAC likely connects to the DFG area or to Ser783, a crucial amino acid that affects the selectivity between HER2 and EGFR activities (Figure 12) (Section 2.2.6).

### 2.4. Virtual Screening

In silico screening supports the high-throughput evaluation of a large number of potential candidate molecules. This computational method for prioritizing initial virtual candidates is commonly referred to as virtual screening (VS). Historically, VS campaigns have focused either on the ligands or the receptors [2]. Specifically, in the context of ligand-based VS, methods that utilize the three-dimensional structural details of small molecules, such as pharmacophore searches, often yield a more comprehensive understanding of biological mechanisms than 2D descriptors. The objective is to engage in scaffold hopping, which entails discovering new active compounds that may possess entirely distinct chemical structures (scaffolds) from the known active compounds while still retaining the same crucial binding characteristics.

In this research, the pharmacophore models identified earlier were employed in ligand-based virtual screening of the ChEMBL34 library, applying particular parameters outlined in the materials and methods (Section 3.4). The outcomes were then integrated into receptor-based virtual screening (molecular docking—validation) for further assessment, according to the criteria mentioned earlier (Section 2.2.1).

#### 2.4.1. Vascular Endothelial Growth Factor Receptor 2 (VEGFR-2)

Fourteen out of twenty-six compounds are successfully identified as meeting the pharmacophore model’s requirements in molecular docking studies (Table 6).

Eight out of fourteen compounds displayed at least one hydrogen bond with the newly identified residue Asn923, often described as part of the solvent-accessible region [33], contributing to the overall stability and specificity of the ligand–protein complex. The most promising compounds, ChEMBL4790167 {2}, ChEMBL4171108 {4}, ChEMBL3661578 {7}, ChEMBL3641531 {8}, and ChEMBL2170947 {10}, surpassed all soft thresholds for affinity values and established all basic hydrophilic (e.g., Cys919) and hydrophobic interactions (e.g., Val848, Val916).

#### 2.4.2. Platelet-Derived Growth Factor Receptor Alpha (PDGFRα)

The application of our stringent criteria during the ChEMBL virtual screening resulted in the identification of a single compound as a potent PDGFRα inhibitor (Table 7).

Compound ChEMBL5019511 established a fundamental hydrogen bond with the hinge region (Cys677) and exhibited several basic hydrophobic interactions (Val607, Val658). These interactions contributed to a favorable CNN affinity; however, the compound fell short of the CNN pose score soft threshold.

#### 2.4.3. Epidermal Growth Factor Receptor (EGFR)

ChEMBL’s virtual screening which was conducted under specific parameters identified eighteen compounds. Nonetheless, only nine were able to meet the molecular docking criteria (Table 8).

It should be noted that the new affinity threshold was established at −7.95, in accordance with Section 2.2.5, and all compounds exceeded this affinity limit. However, only two compounds, ChEMBL4865595 {2} and ChEMBL2216869 {6}, were able to satisfy all three soft thresholds at the same time. Notably, ChEMBL2216869 did not have any interactions with the hinge region but demonstrated the most promising outcomes. One possible explanation for this could be the formation of a covalent bond with Cys797, which is situated at the edge of the active site cleft and recognized as the most solvent-exposed cysteine in the EGFR kinase domain.

#### 2.4.4. Receptor Tyrosine–Protein Kinase erbB-2 (HER2)

Pharmacophore-based virtual screening initially yielded eight candidate compounds. However, the subsequent molecular docking analyses served as a critical filter, approving only a single compound (Table 9).

Compound ChEMBL3355044 exhibited known hydrophilic and hydrophobic interactions with key areas (hinge, DGF motif), resulting in favorable affinity values.

## 3. Materials and Methods

### 3.1. Molecular Similarity

The Tanimoto coefficient is an association metric tailored for binary data, where 0 and 1 signify the absence and presence of molecular structures, respectively. It is calculated as the number of common features shared by both structures (*c*) divided by the total number of features present in either structure (*a* and *b*) minus the common features (*c*) [16].


(1) $$\mathrm{Tani}=\frac{c}{a+b-c\;}$$

To quantify the similarity of molecular representations using the Tanimoto coefficient, we obtained atom pair (AP) and Molecular ACCess Systems keys (MACCS) fingerprints through the online platforms, ChemDes [34,35] and ChemMine [36,37]. A molecular fingerprint provides a way to characterize a molecular structure by converting it into a bit string. As molecular fingerprints encode a molecule’s structure, they serve as an effective method for describing structural similarities among different molecules. Typically, there are two approaches to describe a molecular structure using fingerprints: substructure key-based fingerprints and topological path-based fingerprints [38]. Substructure key-based fingerprints encode the presence of predefined structural features in a molecule, such as MACCS fingerprint. In contrast, topological path-based fingerprints, like the Atom Pairs fingerprint (AP), represent atom connectivity patterns.

### 3.2. Molecular Docking

In molecular docking research, X-ray crystal structures were obtained from the Protein Data Bank via the Research Collaboration for Structural Bioinformatics (RCSB) website [39]. Protein preparation was carried out using OpenMM v8.1.2 [40,41], where energy minimizations were performed utilizing either the AMBER14 [42] or Charm36 [43] force fields. Ligand 3D coordinates were generated and minimized using GypSUm-DL v1.2.1 [44,45], while docking was performed with GNINA v1.0 [46,47], a molecular docking program that integrates convolutional neural networks (CNNs) for scoring and optimizing ligands.

The docking studies were conducted for enhanced accuracy, by enabling flexibility solely in the side chains within 3.5 Å of the co-crystallized ligand. Furthermore, we utilized the built-in CNN_crossdock_default2018_dense_3 model, which employs an architecture of 3D convolutional and pooling layers followed by two distinct fully connected output layers for pose scoring and affinity prediction. This model was trained on the PDBbind v2016 and CrossDocked2020 datasets using custom fork of the Caffe deep learning framework [48]. Under the CNN_scoring rescoring protocol, the CNN model was applied to rank ligand conformations only during the final sorting stage, following initial refinement via empirical scoring function [46]. The min_RMSD_filter was set to 1 to ensure that the resulting conformations vary more than the specified threshold. Input files for docking were visualized using PyMOL v3.0.4 [49] and Schrödinger Maestro v14.5.131 [50].

To validate the protocols associated with molecular docking studies (which include re-docking and cross-docking), we employed Python v3.2.2 [51] to identify matching atoms between docked and co-crystallized ligands and calculate the RMSD values. In terms of verifying the docking software, the main objective was to utilize active structures and decoys obtained from publicly available repositories [52,53], and compute the enrichment factor using the formula below [2]:

(2) $${\mathrm{EF}}_{x\%}=\frac{\frac{{\mathrm{actives}}_{x\%}}{{\mathrm{dataset}}_{x\%}}}{\frac{{\mathrm{actives}}_{\mathrm{total}}}{{\mathrm{dataset}}_{\mathrm{total}}}}$$

where *actives_x%_* refers to the active compounds present in the selected dataset (*dataset_x%_*), while *dataset_total_* encompasses all compounds within that dataset, and *actives_total_* indicates the number of active molecules included among the decoys. We defined *x%* as 5%, which means we aimed to determine how many active compounds exist within the top 5% of our ranked dataset. Additionally, ROC-AUCs were created using Python.

### 3.3. Pharmacophore Modeling

We employed the ChEMBL database to identify all small molecules classified as approved drugs or clinical candidates associated with specific biological targets. After converting these compounds into a 3D format using GypSUm-DL v1.2.1, we examined their interactions with the receptors through molecular docking studies (initial), as described in Section 3.2. Only those that met our previously mentioned criteria (Section 2.2.1) were merged with the notable compounds from the preliminary docking findings (Figure 1). Datasets for each biological target were compiled, and pharmacophore modeling was performed using LigandScout v4.5 [54,55] to pinpoint the essential groups responsible for inhibiting biological responses.

### 3.4. Virtual Screening

For the pharmacophore-based virtual screening of ChEMBL34, we employed Pharmit webserver [56,57], which offers an online and interactive platform for screening large compound databases via pharmacophores and molecular shapes. Pharmacophore queries were initiated using pharmacophore files formatted for LigandScout v4.5, illustrating features such as hydrogen bond acceptors and donors, negative and positive charges, aromatic structures, and hydrophobic characteristics. A pharmacophore/shape search was conducted where the chosen database was first examined for compounds that fit the defined pharmacophore. Subsequently, shape constraints (tolerance 1.5) were applied to the pharmacophore-aligned poses to remove compounds that, while matching the pharmacophore, would cause significant steric clashes with the receptor. Furthermore, compounds were filtered by applying specific ranges derived from FDA-approved drugs identified in our earlier study [6]. These parameters were set as follows: **MW [416.81, 461.47]**, **TPSA [83.48, 95.83]**, **LogPo/w [2.62, 3.49]**, **nRB [5, 7]**, **nHA [4, 10]**, **nHD [0, 5]**, and **nRings [4, 5]**. Finally, results were aligned to the pharmacophore and ranked based on the root mean squared deviation (RMSD) between the features of the query and those of the hit compounds. Ultimately, an energy minimization was performed on the results to refine both the pose and conformation of the identified hits concerning the provided receptor, using GNINA v1.0, parameterized as described in the molecular docking studies (validation).

## 4. Conclusions

Compounds **TKI.2a**, **TKI.2b**, **TKI.6**, **TKI.16**, **TKI.19**, and **TKI.21b**, which were prominent in our earlier research [6], indicated novel biological targets through studies on molecular similarities. To confirm these findings, preliminary molecular docking studies were carried out using soft (affinity: −9.00, CNN pose score: 0.843, CNN affinity: 7.702) and hard (affinity: −6.82, CNN pose score: 0.526, CNN affinity: 7.010) thresholds determined through statistical analyses of molecular docking outcomes related to FDA-approved drugs and their biological targets. Additionally, the interactions between the chemical groups of the compounds and the receptors were also considered. Consequently, compounds **TKI.2a**, **TKI.2b**, **TKI.6**, and **TKI.19** appear to exhibit multi-target tyrosine kinase inhibitory activity against VEGFR-2, RET, PDGFRα, EGFR, and HER2 receptors.

Upon integrating the initial molecular docking results of ChEMBL’s compounds targeting VEGFR-2, PDGFRα, EGFR, and HER2 proteins, we proceeded with pharmacophore modeling studies. From all the pharmacophore models, it can be concluded that the identified hydrogen bond acceptors (HACs) and their unique spatial configurations are crucial for interaction with the hinge and DFG areas of the receptors. Moreover, hydrophobic and aromatic pharmacophoric groups are essential for engaging with significant hydrophobic residues and selectivity pockets. These characteristics seem vital for obstructing the receptor and, consequently, inhibiting the biological response.

Finally, pharmacophore models were applied for ligand-based virtual screening using defined parameters to discover candidate compounds that exhibit drug-likeness with FDA-approved tyrosine kinase inhibitors. The structure-based virtual screening (molecular docking studies) identified lead compounds for each biological target based on their overall affinity values and established interactions. Compound **ChEMBL2170947**, originally designed as a c-Met inhibitor [58], emerged as the most promising candidate for **VEGFR-2** due to its unique dual interactions with the hinge region; its CNN-based parameters were only marginally inferior to those of Tivozanib. **ChEMBL5019511**, initially screened for antimalarial therapy [59], was identified as a lead for **PDGFRα**; however, it exhibited a significantly worse CNN pose score than Imatinib. Furthermore, **ChEMBL2216869**—a potent PI3Kδ (Phosphoinositide 3-kinase delta) inhibitor developed for autoimmune diseases [60]—was selected for **EGFR**, as its binding parameters exceeded the average values of the FDA-approved inhibitors for this protein. Finally, **ChEMBL3355044**, originally synthesized as a PERK (PKR-like endoplasmic reticulum kinase) inhibitor [61], was identified for **HER2** after demonstrating a superior CNN pose score compared to the covalent co-crystallized inhibitor (Table 2 and Table S2).

In future studies, we aim to experimentally validate the potential of compounds **TKI.2a**, **TKI.2b**, **TKI.6**, **TKI.19**, **ChEMBL2170947**, **ChEMBL5019511**, **ChEMBL2216869**, and **ChEMBL3355044** to inhibit the receptors under question. Furthermore, in terms of repositioning, we will apply pharmacophore modeling studies to identify existing synthesized compounds that may inhibit tyrosine kinase receptors, even if they were not originally designed for that purpose. Repurposing methodology is efficient in terms of time and cost, promoting sustainability.

## Figures and Tables

**Table 1:** Verification of primary biological targets of compounds following molecular docking analyses through our novel screening approach.

No.	Compound	Structure	Reported Biological Target	Reference
**1**	**TKI.2a**	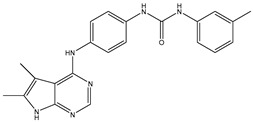	VEGFR-2 (Vascular Endothelial Growth Factor Receptor)	[7]
**2**	**TKI.2b**	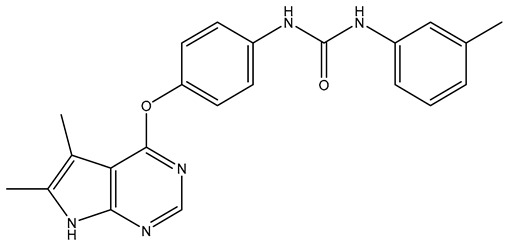	VEGFR-2	
**3**	**TKI.6**	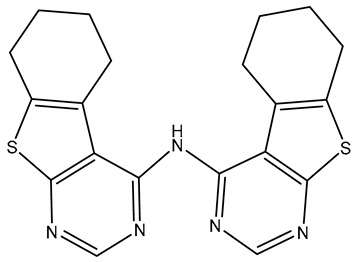	Dual EGFR/HER2 (Epidermal Growth Factor Receptor/Human Epidermal Receptor 2)	[8]
**4**	**TKI.16**	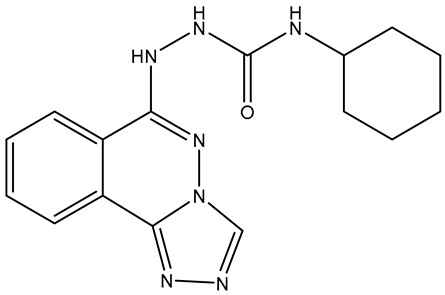	VEGFR-2	[9]
**5**	**TKI.19**	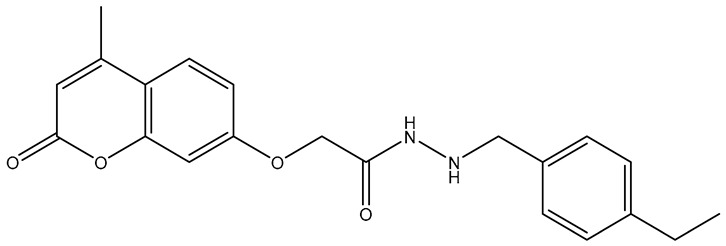	VEGFR-2	[10]
**6**	**TKI.21b**	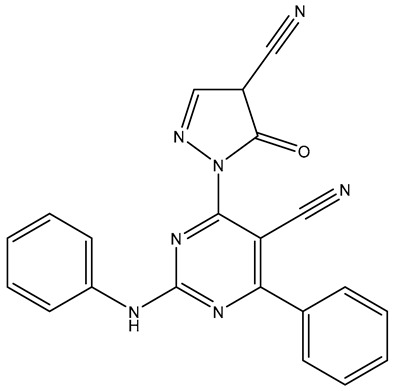	EGFR	[11]

**Table 2:** Compounds displaying their updated biological targets following molecular similarity analyses using the Tanimoto coefficient.

Compound	Reported Biological Target	Targeted Kinases Identified by Molecular Similarity Studies ^1^	Drug Exhibiting the Maximum Tanimoto Index	Structure
**TKI.2a**	VEGFR-2	HER2, c-Kit (SCFR—Stem Cell Factor Receptor), PDGFRα, MEK1/2 (Mitogen-Activated Protein Kinase Kinase), **VEGFR-2**	Tivozanib (VEGFR-2)	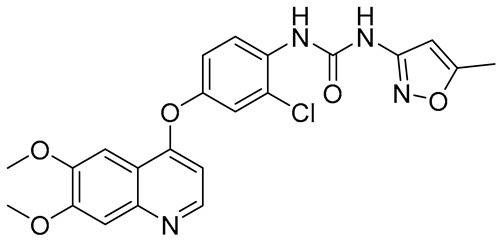
**TKI.2b**	VEGFR-2	**VEGFR-**1/**2**/3, RET (proto-oncogene tyrosine–protein kinase receptor), HER2, c-Kit (SCFR), PDGFRα, MEK1/2	Tivozanib (VEGFR-2)	
**TKI.6**	dual EGFR/HER2	-	-	-
**TKI.16**	VEGFR-2	**JAK1**/2 (Janus kinase)	Filgotinib (JAK1)	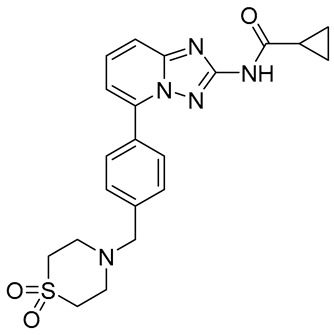
**TKI.19**	VEGFR-2	-	-	-
**TKI.21b**	EGFR	**c-MET (HGFR)**	Capmatinib (c-MET/HGFR)	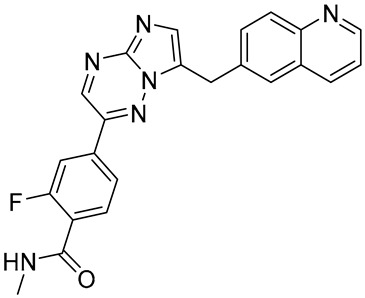

^1^ Bold = highest Tanimoto index; color index: green = reported biological target.

**Table 3:** Statistical analyses of molecular docking data for FDA-approved drugs and their biological targets.

Biological Target	Drug	Affinity (kcal/mol)	CNN Pose Score	CNN Affinity	Mean Affinity (kcal/mol)	Mean CNN Pose Score	Mean CNN Affinity
VEGFR−2	Axitinib	−8.53	0.842	7.634	−9.77	0.882	7.765
	Cabozatinib	−11.86	0.913	7.725			
	Fruquitinib	−8.74	0.906	7.672			
	Lenvatinib	−11.03	0.957	8.049			
	Pazopanib	−8.69	0.856	7.407			
	Regorafenib	−11.24	0.890	7.833			
	Sorafenib	−11.25	0.882	7.588			
	Sunitinib	−7.35	0.728	7.312			
	Tivozanib	−10.94	0.946	8.408			
	Vandetanib	−8.03	0.900	8.025			
PDGFRα	Avapritinib	−6.82	0.904	8.235	−7.87	0.892	8.079
	Ripretinib	−8.91	0.881	7.922			
c−MET	Capmatinib	−11.41	0.901	7.976	−10.13	0.912	8.129
	Tepotinib	−10.00	0.863	8.222			
	Savolitinib	−8.99	0.973	8.189			
RET	Cabozatinib	−8.83	0.526	7.169	−9.00	0.820	7.572
	Lenvatinib	−7.24	0.927	7.532			
	Pralsetinib	−10.09	0.974	8.053			
	Selpercatinib	−9.83	0.854	7.535			
EGFR	Afatinib	−8.35	0.900	7.852	−7.95	0.945	8.027
	Dacomitinib	−8.60	0.932	8.125			
	Gefitinib	−7.93	0.983	7.986			
	Mobocertinib	−7.77	0.979	8.225			
	Osimertinib	−7.12	0.932	7.948			
HER2	Afatinib	−7.61	0.925	7.381	−9.09	0.842	7.590
	Capivasertib	−9.71	0.898	7.450			
	Lapatinib	−9.98	0.858	7.609			
	Neratinib	−7.51	0.780	7.875			
	Tucatinib	−10.64	0.750	7.634			
JAK1	Abrocitinib	−9.10	0.974	7.630	−9.03	0.847	7.611
	Ruxolitinib	−9.05	0.943	7.976			
	Filgotinib	−8.12	0.586	7.010			
	Upadacitinib	−9.85	0.886	7.826			
JAK2	Fedratinib	−7.83	0.964	8.352	−8.47	0.938	7.856
	Momelotinib	−8.71	0.967	7.799			
	Pacritinib	−9.22	0.945	7.551			
	Ruxolitinib	−8.02	0.910	7.796			
	Baricitinib	−8.55	0.902	7.780			
BTK	Acalabrutinib	−11.44	0.843	7.959	−10.44	0.852	7.607
	Ibrutinib	−9.80	0.858	7.688			
	Pirtobrutinib	−10.01	0.755	7.189			
	Zanubrutinib	−10.50	0.954	7.593			
BCR−Abl	Asciminib	−10.69	0.797	7.737	−10.38	0.762	7.771
	Bosutinib	−8.80	0.748	7.687			
	Dasatinib	−9.84	0.803	7.527			
	Imatinib	−11.22	0.638	7.613			
	Nilotinib	−10.72	0.833	8.306			
	Ponatinib	−11.00	0.755	7.758			
Hard Thresholds	Mean	−9.32	0.867	7.778			
	Minimum	−11.86	0.526	7.010			
	Maximum	−6.82	0.983	8.408			

**Table 4:** Compounds displayed their verified biological targets following molecular docking analyses in relation to those identified through molecular similarity studies.

Compound	Reported Biological Target	Targeted Kinases Identified by Molecular Similarity Studies ^1^	Targeted Kinases Verified by Molecular Docking Studies ^1^
**TKI.2a**	VEGFR-2	HER2, c-Kit/SCFR, PDGFRα, MEK1/2, **VEGFR-2**	RET, PDGFRα, EGFR, HER2
**TKI.2b**	VEGFR-2	**VEGFR-**1/**2**/3, RET, HER2, c-Kit/SCFR, PDGFRα, MEK1/2	PDGFRα, HER2, c-MET
**TKI.6**	dual EGFR/HER2	-	VEGFR-2
**TKI.16**	VEGFR-2	**JAK1**/2	-
**TKI.19**	VEGFR-2	-	PDGFRα, EGFR, c-MET
**TKI.21b**	EGFR	**c-MET/HGFR**	-

^1^ Bold = highest Tanimoto index, color index: green = reported biological target, blue = biological target identified by molecular similarity studies.

**Table 5:** Results from cross-docking demonstrating affinity, CNN pose score, CNN affinity, and RMSD values for known drugs pertaining to each biological target.

Target	Drug	Affinity (kcal/mol)	CNN Pose Score	CNN Affinity	Cross-Docking RMSD (Å)
**VEGFR-2**	Axitinib	−8.53	0.842	7.634	5.500
	Cabozatinib	−11.86	0.913	7.725	1.059
	Fruquitinib	−8.74	0.906	7.672	1.499
	Lenvatinib	−11.03	0.957	8.049	2.776
	Pazopanib	−8.69	0.856	7.407	3.730
	Regorafenib	−11.24	0.890	7.833	1.688
	Sorafenib	−11.25	0.882	7.588	2.536
	Sunitinib	−7.35	0.728	7.312	5.250
	Vandetanib	−10.42	0.814	8.062	1.514
**RET**	Cabozatinib	−8.83	0.526	7.169	1.920
	Lenvatinib	−7.24	0.927	7.532	3.108
	Selpercatinib	−9.83	0.854	7.535	2.064
**PDGFRα**	Avapritinib	−6.82	0.904	8.235	5.095
	Ripretinib	−8.91	0.881	7.922	2.000
**EGFR**	Afatinib	−8.35	0.900	7.852	2.169
	Dacomitinib	−8.60	0.932	8.125	2.186
	Gefitinib	−7.93	0.983	7.986	1.862
	Osimertinib	−7.12	0.932	7.948	1.562
**HER2**	Afatinib	−7.61	0.925	7.381	2.906
	Capivasertib	−9.71	0.898	7.45	2.537
	Lapatinib	−9.98	0.858	7.609	2.022
	Neratinib	−7.51	0.780	7.875	2.432
	Tucatinib	−10.64	0.750	7.634	1.494
**c-MET**	Capmatinib	−11.41	0.901	7.976	2.534
	Savolitinib	−8.99	0.973	8.189	1.079

**Table 6:** Compounds that fulfill molecular docking criteria following the virtual screening of ChEMBL34, according to the VEGFR-2 pharmacophore model.

No.	Compound	Structure	Affinity	CNN Pose Score	CNN Affinity	Interactions
**1**	**ChEMBL** **3661566**	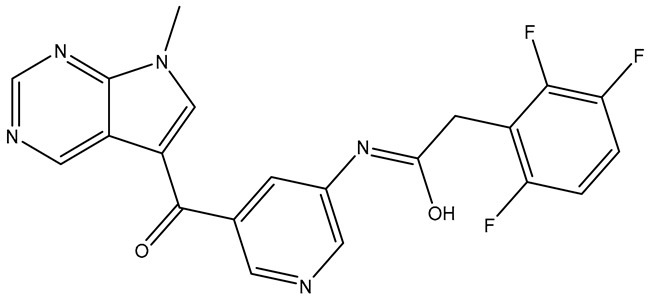	−10.99	0.537	7.802	Hydrophobic: Val840, Val848, Leu889, Val898, Val899, Val916/Hydrogen bonds: Asp1046
**2**	**ChEMBL** **4790167**	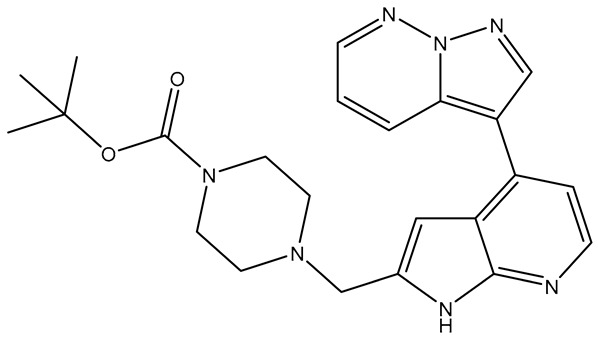	−10.28	0.875	7.894	Hydrophobic: Val848, Ile888, Val898, Val899, Val916, Leu1019, Leu1019, His1026, Leu1035/Hydrogen bonds: Cys919, Asp1046
**3**	**ChEMBL** **3661571**	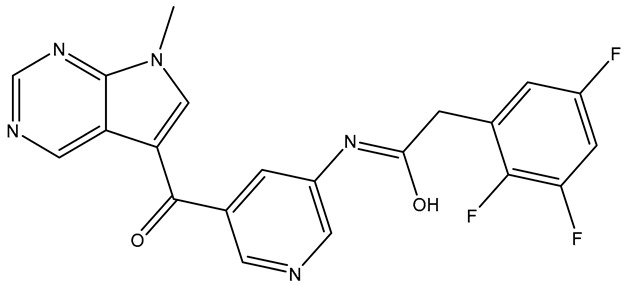	−10.44	0.804	7.759	Hydrophobic: Val840, Val840, Val840, Val848, Val848, Lys868, Leu889, Leu1035/Hydrogen bonds: Asn923, Asn923
**4**	**ChEMBL** **4171108**	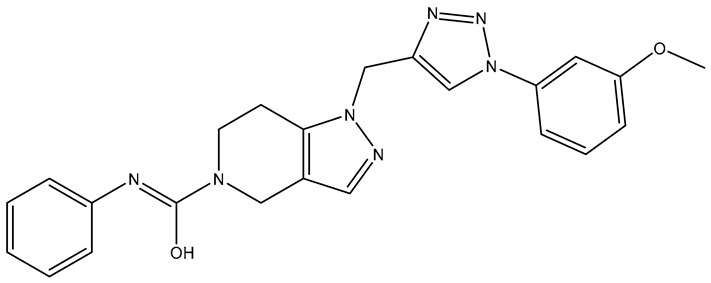	−10.14	0.896	8.176	Hydrophobic: Val840, Val848, Val848, Leu889, Val899, Val916, Phe918, Asp1046/Hydrogen bonds: Glu885, Cys919, Asn923, Asn923/π-stacking: Phe1047
**5**	**ChEMBL** **2354367**	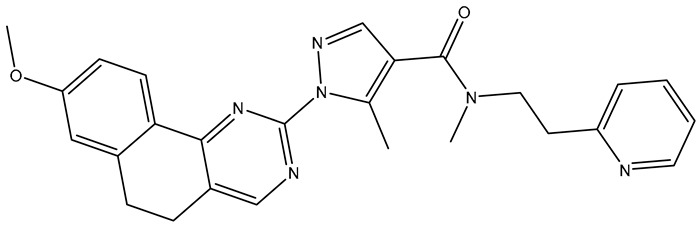	−10.14	0.809	8.386	Hydrophobic: Val840, Val840, Val840, Val848, Glu885, Leu889, Val899, Val916, Phe918, Asp1046, Phe1047/Hydrogen bonds: Cys919, Asn923, Asp1046
**6**	**ChEMBL** **4581299**	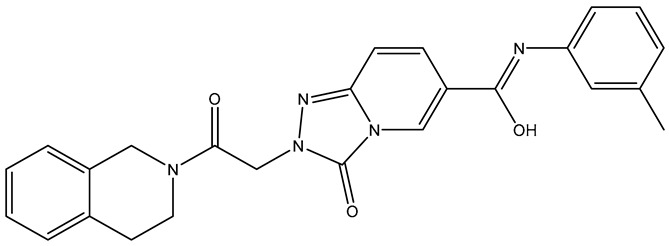	−11.12	0.879	7.467	Hydrophobic: Lys838, Val840, Val848, Lys868, Leu889, Leu889, Val914, Val916, Phe918, Phe1047/Hydrogen bonds: Cys919, Asn923
**7**	**ChEMBL** **3661578**	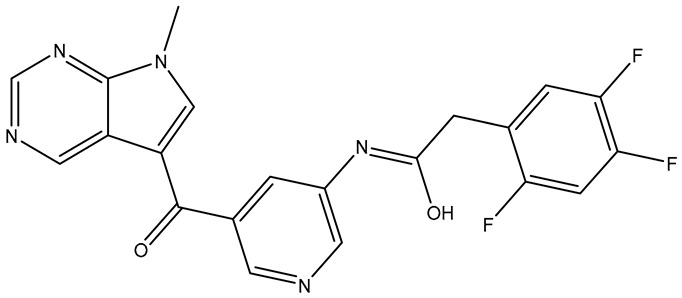	−10.52	0.8671	7.804	Hydrophobic: Val840, Val840, Lys868, Val899, Val916, Val916, Leu1035/Hydrogen bonds: Cys919, Asn923
**8**	**ChEMBL** **3641531**	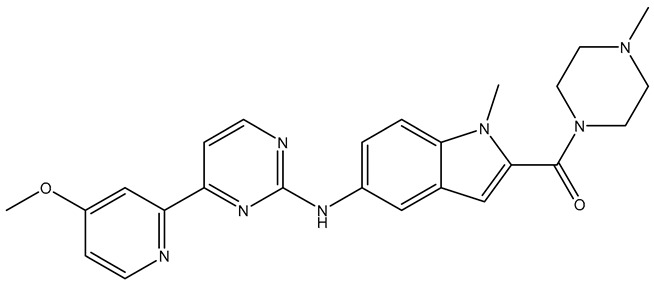	−10.85	0.905	8.133	Hydrophobic: Val840, Val848, Val899, Val916, Phe918, Leu1035, Phe1047/Hydrogen bonds: Glu917, Cys919, Asn923, Asn923
**9**	**ChEMBL** **4092441**	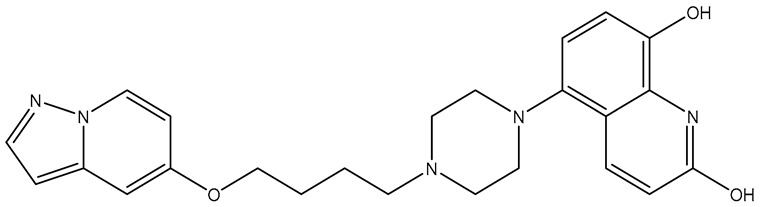	−9.55	0.7488	7.734	Hydrophobic: Lys868, Leu882, Glu885, Glu885, Ile888, Leu889, Val898, Asp1046/Hydrogen bonds: Glu885, Asp1046, Phe1047
**10**	**ChEMBL** **2170947**	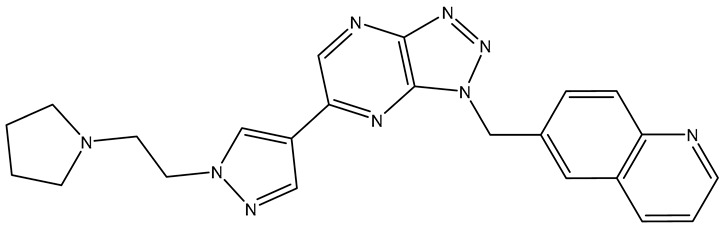	−9.11	0.878	8.273	Hydrophobic: Val848, Val848, Val899, Val916, Val916, Phe1047/Hydrogen bonds: Glu917, Cys919, Cys919, Asn923/π-stacking: Phe1047
**11**	**ChEMBL** **3661581**	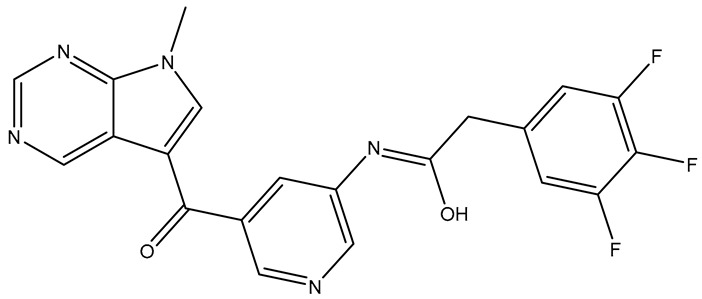	−10.03	0.763	8.067	Hydrophobic: Val848, Leu889, Ile892, Asp1046/Hydrogen bonds: Asp1046/π-stacking: Phe1047
**12**	**ChEMBL** **1459733**	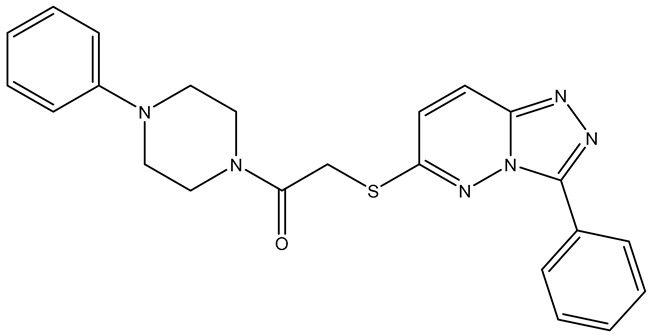	−9.98	0.922	7.120	Hydrophobic: Leu840, Val848, Val899, Val916, Leu1035, Arg1051, Tyr1059/Hydrogen bonds: Cys919
**13**	**ChEMBL** **3661565**	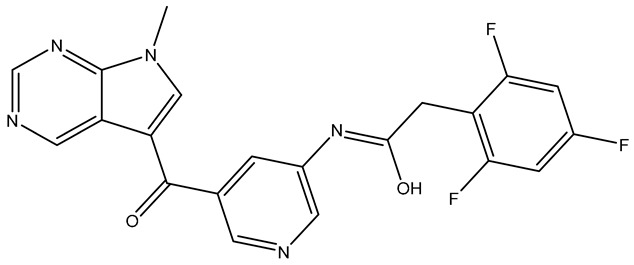	−10.82	0.800	7.989	Hydrophobic: Val840, Glu885, Leu889, Leu889, Val916, Leu1035, Leu1035/Hydrogen bonds: Cys919, Asn923
**14**	**ChEMBL** **3318995**	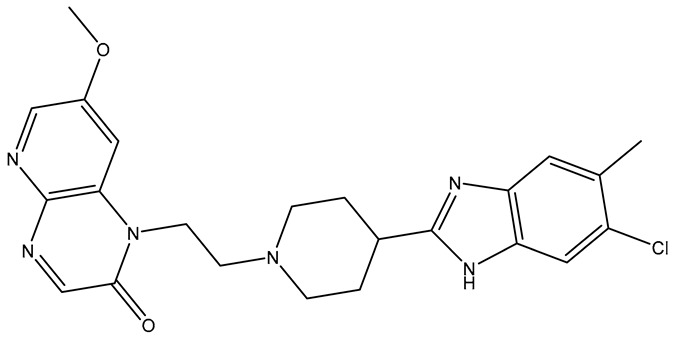	−10.11	0.597	7.962	Hydrophobic: Val840, Val840, Leu889, Val898, Val899, Val916, Leu1019, Phe1047/Hydrogen bonds: Lys868, Cys919, Asp1046

**Table 7:** Compounds that fulfill molecular docking criteria following the virtual screening of ChEMBL34, according to the PDGFRα pharmacophore model.

No.	Compound	Structure	Affinity	CNN Pose Score	CNN Affinity	Interactions
**1**	**ChEMBL** **5019511**	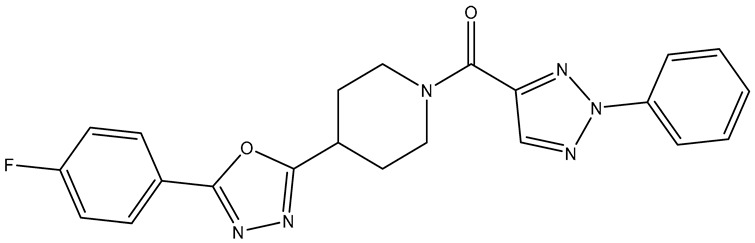	−10.36	0.804	7.725	Hydrophobic: Leu599, Leu599, Leu599, Val607, Lys627, Val658, Tyr676, Asp836, Phe837/Hydrogen bonds: Cys677

**Table 8:** Compounds that fulfill molecular docking criteria following the virtual screening of ChEMBL34, according to the EGFR pharmacophore model.

No.	Compound	Structure	Affinity	CNN Pose Score	CNN Affinity	Interactions
**1**	**ChEMBL** **3903973**	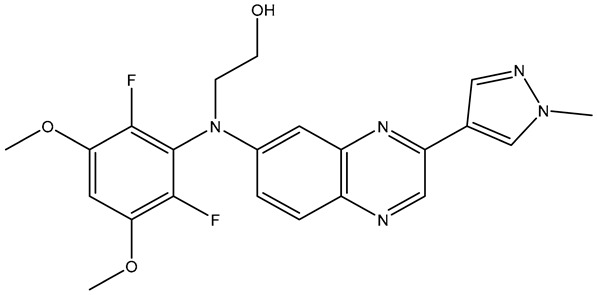	−8.06	0.791	7.780	Hydrophobic: Leu718, Ala743/Hydrogen bonds: Met793, Cys797
**2**	**ChEMBL** **4865595**	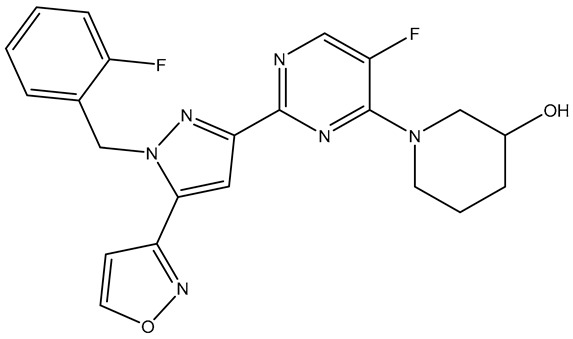	−8.11	0.880	7.755	Hydrophobic: Leu718, Val726, Lys745, leu788, Thr790, Arg841, Leu844, Thr854/Hydrogen bonds: Met793, Asp800, Asp855
**3**	**ChEMBL** **59202**	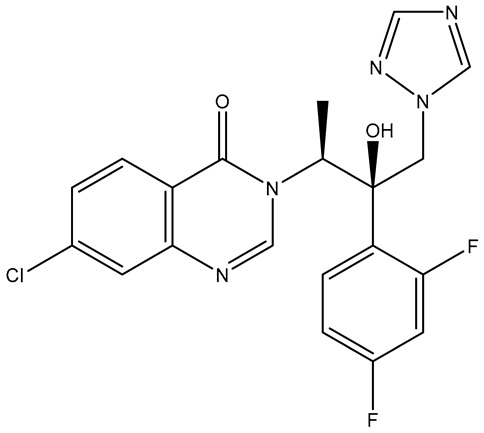	−8.69	0.587	7.696	Hydrophobic: Leu718, Val726, Ala743, Leu844/Hydrogen bonds: Lys745, Thr854, Asp855, Asp855/π-stacking: Phe723
**4**	**ChEMBL** **3657549**	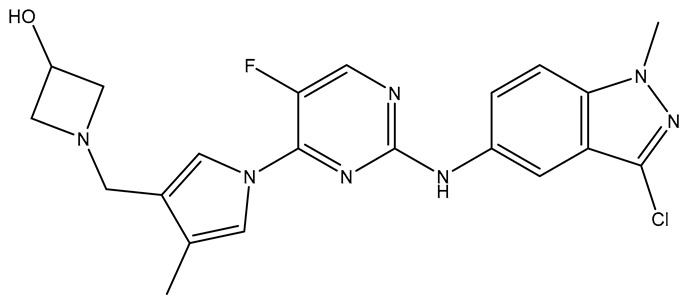	−8.62	0.843	7.286	Hydrophobic: Leu718, Val726, Phe723/Hydrogen bonds: Met793
**5**	**ChEMBL** **3984043**	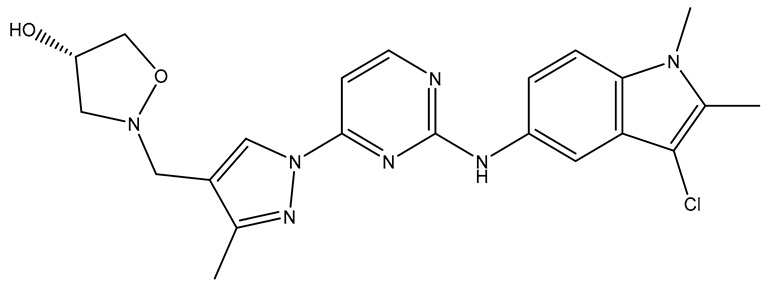	−8.49	0.890	7.441	Hydrophobic: Leu718, Phe723, Val726, Val726, Val726, Leu844/Hydrogen bonds: Met793, Met793, Asp800
**6**	**ChEMBL** **2216869**	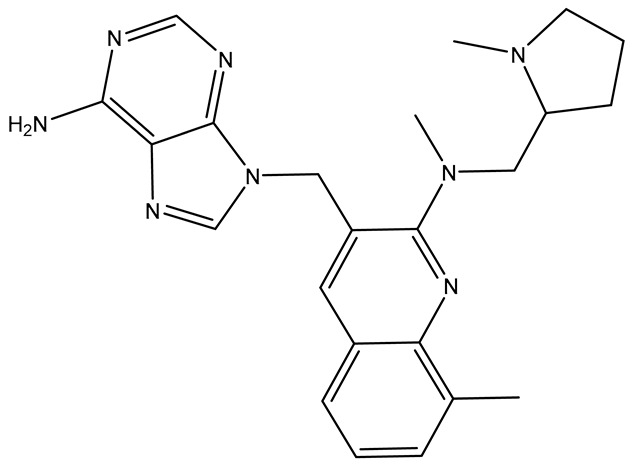	−9.00	0.966	8.171	Hydrophobic: Leu718, Leu718, Leu718, Phe723/Hydrogen bonds: Thr790, Gln791, Thr854
**7**	**ChEMBL** **165023**	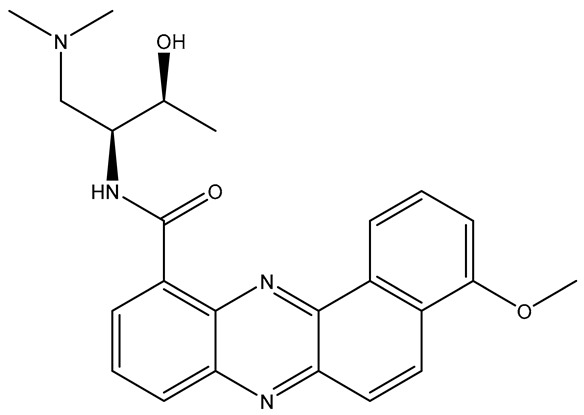	−8.49	0.889	7.323	Hydrophobic: Leu718, Leu718, Val726, Ala743, Met793, Arg841, Leu844/Hydrogen bonds: Thr790
**8**	**ChEMBL** **5091998**	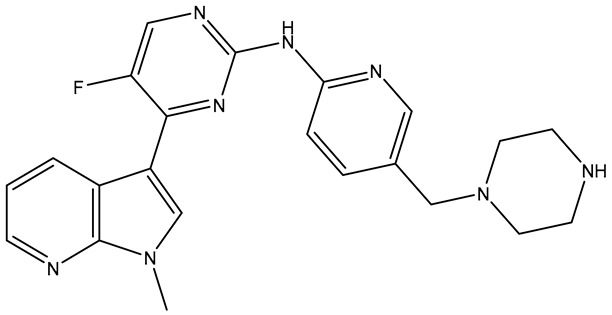	−8.00	0.911	7.268	Hydrophobic: Leu718, Val726, Leu844, Leu844/Hydrogen bonds: Met793, Met793
**9**	**ChEMBL** **2041238**	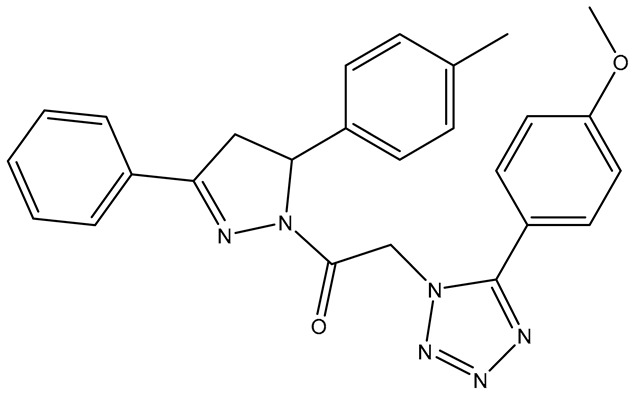	−9.05	0.835	7.528	Hydrophobic: Leu718, Leu718, Phe723, Val726, Ala743, Leu844, Thr854/Hydrogen bonds: Lys745, Met793, Thr854, Asp855

**Table 9:** Compounds that fulfill molecular docking criteria following the virtual screening of ChEMBL34, according to the HER2 pharmacophore model.

No.	Compound	Structure	Affinity	CNN Pose Score	CNN Affinity	Interactions
**1**	**ChEMBL** **3355044**	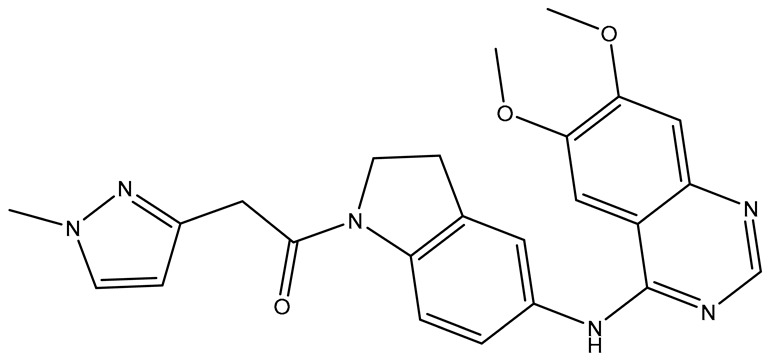	−10.39	0.869	7.564	Hydrophobic: Leu726, Phe731, Lys753, Thr798, Met801, Thr862, Asp863, Phe864/Hydrogen bonds: Lys753, Met801, Asp863, Phe864

## Data Availability

The data are available by the authors and through literature.

## Supplementary Materials

The following supporting information can be downloaded at https://www.mdpi.com/article/10.3390/molecules31101689/s1, Table S1: Tanimoto’s coefficients of compounds TKI.2a, TKI.2b, TKI.16 and TKI.21b in comparison to various known FDA-approved drugs.; Table S2: Hydrogen bond and hydrophobic interactions with proteins, binding affinity values, CNN pose scores and CNN affinities for each compound per biological target. Green indicates values exceeding the statistical threshold, while red signifies those falling below the thresholds.; Table S3: Compounds in the top 5% ranked according to their CNN affinity for HER2.; Table S4: Compounds in the top 5% ranked according to their CNN pose score for HER2.; Table S5: Compounds in the top 5% ranked according to their CNN affinity for VEGFR-2.; Table S6: Compounds in the top 5% ranked according to their CNN pose score for VEGFR-2.
