# Supplementary material for: In Silico Studies of Potent Tyrosine Kinase Inhibitors: Molecular Docking and Pharmacophore Modeling Approaches
Source: Molecules. 2026 May 16;31(10):1689. doi: 10.3390/molecules31101689 (PMC13209961; doi:10.3390/molecules31101689)
Supplement: Supplementary file 1 [file molecules-31-01689-s001.zip › molecules-4232703-supplementary.pdf]

Table S1. Tanimoto's coefficients of compounds TKI.2a, TKI.2b, TKI.16 and TKI.21b in comparison to various known FDA-approved drugs.

Molecule ID TKI.2a

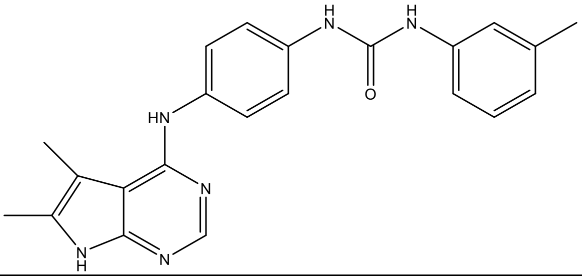

Comparison Drug	Drug Stucture	Similarity method	Fingerprint type Atom pairs	MACCS	Common substructure
**Tucatinib (ErbB2/ HER2)**	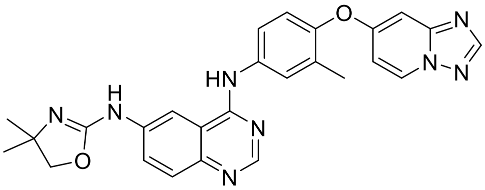	Tanimoto	0.34	0.54	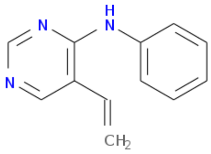
**Ripretinib (Kit, PDGFRα)**	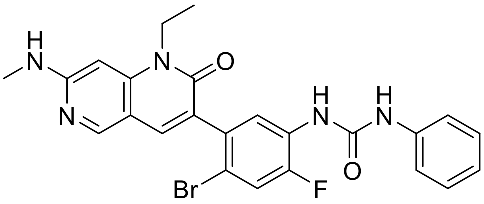	Tanimoto	0.39	0.58	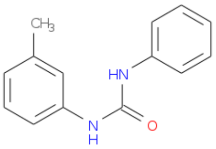
**Trametinib (MEK1/2)**	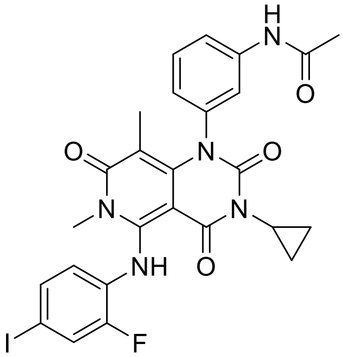	Tanimoto	0.25	0.55	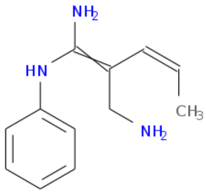
**Tivozanib (VEGFR2)**	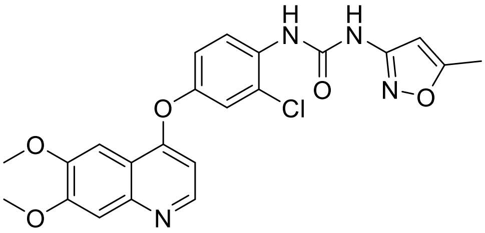	Tanimoto	0.44	0.62	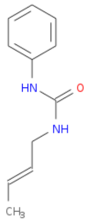

Molecule ID TKI.2b

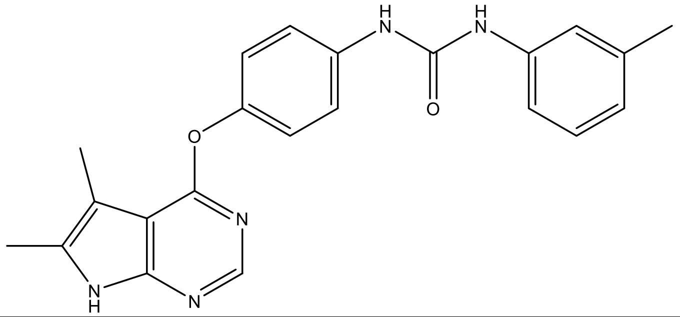

Comparison Drug	Drug Stucture	Similarity method	Fingerprint type<br>Atom pairs	 MACCS	Common substructure
**Lenvatinib (VEGFR1/2/3, RET)**	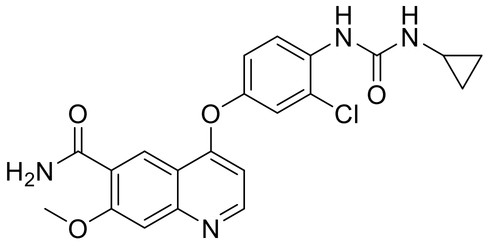	Tanimoto	0.33	0.63	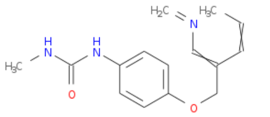
**Tivozanib (VEGFR2)**	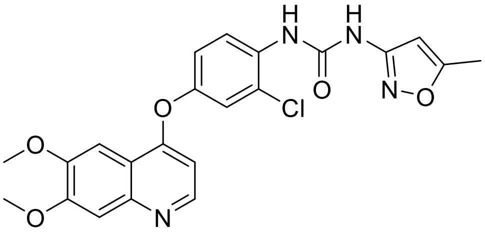	Tanimoto	0.48	0.75	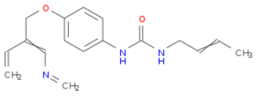
**Cabozantinib (RET, VEGFR2)**	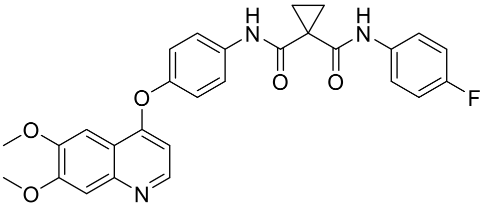	Tanimoto	0.36	0.59	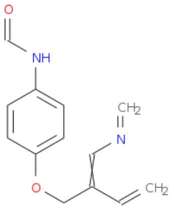
**Tucatinib (ErbB2/ HER2)**	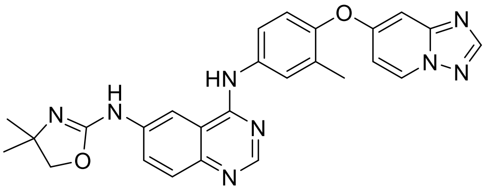	Tanimoto	0.36	0.66	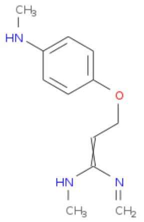
**Ripretinib (Kit, PDGFRα)**	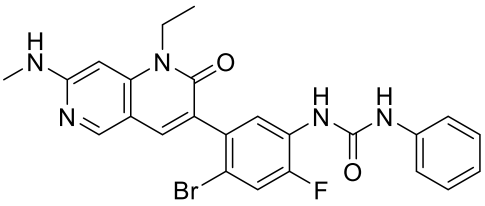	Tanimoto	0.38	0.63	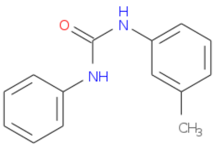

**Trametinib (MEK1/2)**	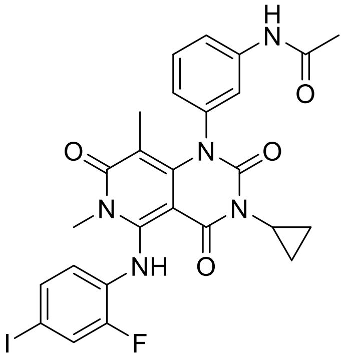	Tanimoto	0.25	0.63	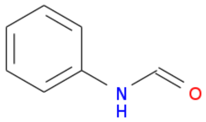

Molecule ID TKI.16

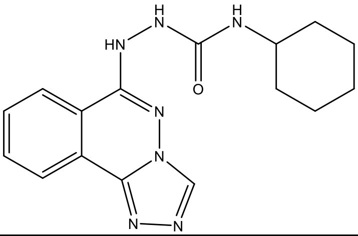

**Comparison Drug**	**Drug Stucture**	**Similarity method**	**Fingerprint type**		**Common substructure**
			**Atom pairs**	**MACCS**	
Ruxolitinib (JAK1/2/3, TYK)	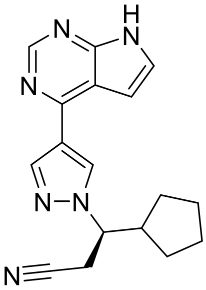	Tanimoto	0.28	0.56	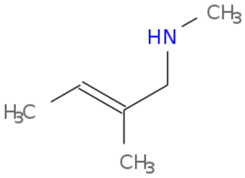
Filgotinib (JAK1)	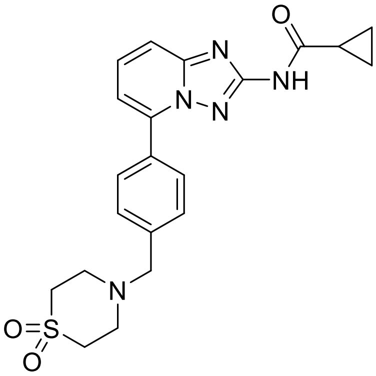	Tanimoto	0.32	0.54	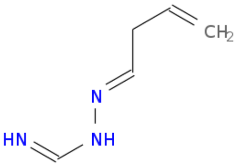

Molecule ID TKI.21b

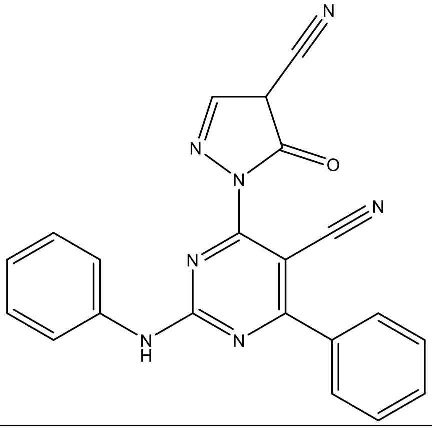

Comparison Drug	Drug Stucture	Similarity method	Fingerprint type		Common substructure
			Atom pairs	MACCS	
**Capmatinib [MET (HGFR)]**	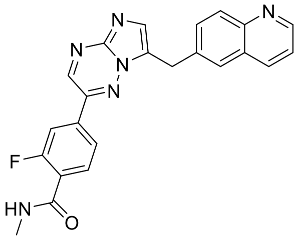	Tanimoto	0.27	0.63	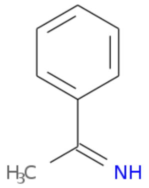

Table S2. Hydrogen bond and hydrophobic interactions with proteins, binding affinity values, CNN pose scores and CNN affinities for each compound per biological target. Green indicates values exceeding the statistical threshold, while red signifies those falling below the thresholds.

**Statistical limits**							**-9,00**	**0,843**	**7,702**
**Target**	**Compound**	**Link 1**	**Link 2**	**Link 3**	**Link 4**	**Hydrophobic interactions**	**Affinity (kcal/mol)**	**CNN pose score**	**CNN affinity**
**VEGFR-2**	**Tivozanib**	Cys919 (hinge)	Asp1046 (DFG motif)	-	-	Leu840, Glu885, Ile888, Leu889, Val899, Val916, Phe918, Leu1035	−10.87	0.925	8.124
	**TKI.6**	Glu885	Leu1049	-	-	Glu885, Val898, Asp1046	−11.13	0.667	7.932
**RET**	**Pralsetinib**	Lys758 (catalytic lysine)	Glu805 (hinge)	Ala807 (hinge)	Ala807 (hinge)	Leu730, Val738, Val804, Leu881, Leu881	−9.89	0.970	8.129
	**TKI.2a**	Leu730	Ala807 (hinge)	Ala807 (hinge)	Asp892 (DFG motif)	Leu730, Leu730, Leu730, Val738, Lys758, Lys758, Leu760	−9.87	0.866	7.349
**PDGFRα**	**Imatinib**	Thr674 (gatekeeper)	Val815	Asp836 (DFG motif)	-	Leu599, Val607, Ala625, Lys627, Lys627, Ile672, Thr674, Leu825, Asp836	−11.05	0.862	7.952

	**TKI.2a**	Lys627	Cys677 (hinge)	Cys677 (hinge)	Asp836 (DFG motif)	Leu599, Leu599, Val607, Glu644, Val658, Leu825, Asp836, Phe837, Phe837, Phe837,	−10.48	0.963	7.936
	**TKI.2b**	Cys677 (hinge)	Asp836 (DFG motif)	-	-	Leu599, Ala625, Lys627, Glu644, Ile647, Val658, Thr674, Leu825, Phe837	−10.59	0.977	8.108
	**TKI.19**	Lys627	Glu644	Cys677 (hinge)	Asp836 (DFG motif)	Leu599, Leu599, Ala607, Ala625, Leu809, Leu809, Asp836	−9.49	0.872	7.040
**EGFR**	**Mobocertinib**	Thr790 (gatekeeper)	Met793 (hinge)	Met793 (hinge)	Thr854	Phe723, Val726, Ala743, Thr790, Leu844	−7.66	0.970	8.106
	**TKI.2a**	Thr790 (gatekeeper)	Met793 (hinge)	Cys797	-	Leu718, Val726, Val726, Asp800, Tyr801, Leu844	−8.60	0.843	7.047
	**TKI.19**	Met793 (hinge)	-	-	-	Phe723, Phe723, Phe723 Val726, Ala743, Leu788, Thr790, Leu844, Leu844, Thr854	−8.17	0.851	7.087
**HER2**	**Inhibitor**	Ser783	Met801 (hinge)	Asp863 (DFG motif)	Phe864 (π–stacking) (DFG motif)	Leu785, Thr798, Thr862	−9.66	0.814	7.734
	**TKI.2a**	Met801 (hinge)	Met801 (hinge)	Asp863 (DFG motif)	Phe864 (π–stacking) (DFG motif)	Leu726, Leu726, Val734, Lys753, Leu796, Leu852, Thr862	−10.71	0.889	7.740
	**TKI.2b**	Met801 (hinge)	Met801 (hinge)	-	-	Leu726, Leu726, Phe731, Val734, Ly753, Ala771, Leu785, Leu796, Leu796, Leu852, Phe864	−10.58	0.885	7.410
**c-MET**	**Tepotinib**	Met1160 (hinge)	Asn1167	Asp1222 (DFG motif)	Tyr1230 (π–stacking)	Val1092, Ala110, Leu1157, Ala1221, Asp1222, Tyr1230, Tyr1230	−10.00	0.863	8.222
	**TKI.2b**	Pro1158	Met1160 (hinge)	Asn1167	-	Val1092, Leu1140, Leu1157, Leu1157, Tyr1230, Asp1231	−9.74	0.843	7.120
	**TKI.19**	Asp1222 (DFG motif)	Tyr1230 (π–stacking)	-	-	Ile1084, Val1092, Ala1108, Leu1157, Met1160, Tyr1230, Asp1231	−9.40	0.856	7.199

Table S3. Compounds in the top 5% ranked according to their CNN affinity for HER2.

	compound_id	true_label	affinity	pose_score	CNN_affinity
1	ZINC13495910	inactive	-8.67	0.9695	8.889
2	BDB27963	active	-10.51	0.9044	8.862
3	BDB27964	active	-9.66	0.8971	8.828
4	BDB27970	active	-8.64	0.9221	8.796
5	BDB5460	active	-10.47	0.9765	8.764
6	ZINC06443991	inactive	-10.00	0.9430	8.735
7	BDB13921	active	-10.83	0.9400	8.726
8	BDB5459	active	-10.03	0.9853	8.663
9	BDB27961	active	-11.93	0.9148	8.632
10	BDB13923	active	-10.88	0.9682	8.543
11	ZINC20911906	inactive	-7.35	0.7011	8.504
12	ZINC13845484	inactive	-7.16	0.8726	8.430
13	BDB5445	active	-11.24	0.9525	8.419
14	BDB5441	active	-10.7	0.9107	8.417
15	ZINC13494871	inactive	-8.31	0.9322	8.368
16	ZINC28132328	inactive	-10.15	0.9226	8.366

Table S4. Compounds in the top 5% ranked according to their CNN pose score for HER2.

	compound_id	true_label	affinity	pose_score	CNN_affinity
1	BDB5459	active	–10.03	0.9853	8.663
2	BDB5447	active	–8.99	0.9816	8.010
3	ZINC08826693	inactive	–10.31	0.9794	8.287
4	BDB5460	active	–10.47	0.9765	8.764
5	ZINC28250348	inactive	–7.84	0.9761	8.144
6	BDB13914	active	–11.49	0.9732	8.297
7	ZINC09282440	inactive	–6.1	0.9724	8.066
8	BDB13902	active	–8.91	0.9713	7.869
9	ZINC19204006	inactive	–9.33	0.9696	8.071
10	ZINC13495910	inactive	–8.67	0.9695	8.889
11	BDB13913	active	–11.29	0.9692	8.186
12	ZINC08435259	inactive	–7.44	0.9690	7.379
13	BDB5446	active	–7.84	0.9687	7.578
14	BDB13923	active	–10.88	0.9682	8.543
15	BDB13911	active	–11.88	0.9664	8.134
16	BDB13900	active	–8.18	0.9641	7.634

Table S5. Compounds in the top 5% ranked according to their CNN affinity for VEGFR-2.

	compound_id	true_label	affinity	pose_score	CNN_affinity
1	CHEMBL389963	active	–10.70	0.9733	8.844
2	CHEMBL230003	active	–10.39	0.9188	8.835
3	CHEMBL230107	active	–10.90	0.9405	8.761
4	CHEMBL427837	active	–10.30	0.9757	8.742
5	CHEMBL462168	active	–9.97	0.9045	8.650
6	CHEMBL231370	active	–10.94	0.919	8.614
7	CHEMBL215943	active	–12.70	0.9779	8.535
8	CHEMBL452569	active	–9.28	0.8608	8.518

9	CHEMBL565807	active	−9.78	0.7918	8.412
10	CHEMBL402531	active	6.70	0.8684	8.36
11	CHEMBL258014	active	13.92	0.9198	8.357
12	CHEMBL459896	active	−9.47	0.9456	8.314
13	CHEMBL212964	active	−12.31	0.9728	8.307
14	CHEMBL238737	active	−9.91	0.9863	8.288
15	CHEMBL411630	active	−9.90	0.8837	8.283
16	CHEMBL189354	active	−11.05	0.8675	8.213
17	CHEMBL270306	active	−7.17	0.8565	8.199
18	CHEMBL195218	active	−7.80	0.7137	8.192
19	CHEMBL219584	active	−12.45	0.9455	8.184
20	CHEMBL409538	active	−9.37	0.8789	8.176
21	CHEMBL231127	active	1.96	0.8666	8.135
22	CHEMBL269915	active	−10.10	0.8879	8.101
23	CHEMBL272691	active	−9.94	0.9132	8.095
24	C65266040	inactive	16.11	0.7937	8.067

Table S6. Compounds in the top 5% ranked according to their CNN pose score for VEGFR-2.

	compound_id	true_label	affinity	pose_score	CNN_affinity
1	CHEMBL238737	active	−9.91	0.9863	8.288
2	C61592421	inactive	−7.11	0.9832	8.031
3	C27564148	inactive	−7.12	0.981	7.112
4	C14124954	inactive	−9.35	0.9797	7.929
5	CHEMBL401040	active	−9.78	0.978	8.038
6	CHEMBL215943	active	−12.70	0.9779	8.535
7	CHEMBL427837	active	−10.30	0.9757	8.742
8	CHEMBL389963	active	−10.70	0.9733	8.844
9	CHEMBL212964	active	−12.31	0.9728	8.307
10	C63743440	inactive	−8.30	0.9717	7.937
11	CHEMBL1173411	active	−10.01	0.9668	7.990
12	CHEMBL220874	active	−12.03	0.9614	7.979
13	C00287606	inactive	−6.54	0.9592	7.047
14	C61630352	inactive	−6.26	0.959	7.089
15	C36948341	inactive	−7.40	0.9572	6.455
16	C06056789	inactive	−7.71	0.9538	7.312
17	C58891869	inactive	−7.49	0.949	7.391
18	CHEMBL459896	active	−9.47	0.9456	8.314
19	CHEMBL219584	active	−12.45	0.9455	8.184
20	CHEMBL376951	active	−9.08	0.9454	8.026
21	C35763652	inactive	−7.77	0.9426	7.259
22	CHEMBL230107	active	−10.90	0.9405	8.761
23	C52839975	inactive	−6.90	0.9394	6.949
24	C37036586	inactive	−6.55	0.9386	7.096
